# Dynamic transcriptome analysis of osteal macrophages identifies a distinct subset with senescence features in experimental osteoporosis

**DOI:** 10.1172/jci.insight.182418

**Published:** 2024-12-06

**Authors:** Yoshio Nishida, M. Alaa Terkawi, Gen Matsumae, Shunichi Yokota, Taiki Tokuhiro, Yuki Ogawa, Hotaka Ishizu, Junki Shiota, Tsutomu Endo, Hend Alhasan, Taku Ebata, Keita Kitahara, Tomohiro Shimizu, Daisuke Takahashi, Masahiko Takahata, Ken Kadoya, Norimasa Iwasaki

**Affiliations:** Department of Orthopedic Surgery, Faculty of Medicine and Graduate School of Medicine, Hokkaido University, Kita-ku, Sapporo, Japan.

**Keywords:** Aging, Immunology, Macrophages, Osteoporosis, p53

## Abstract

Given the potential fundamental function of osteal macrophages in bone pathophysiology, we study here their precise function in experimental osteoporosis. Gene profiling of osteal macrophages from ovariectomized mice demonstrated the upregulation of genes that were involved in oxidative stress, cell senescence, and apoptotic process. A single-cell RNA-Seq analysis revealed that osteal macrophages were heterogeneously clustered into 6 subsets that expressed proliferative, inflammatory, antiinflammatory, and efferocytosis gene signatures. Importantly, postmenopausal mice exhibited an increase in subset 3 that showed a typical gene signature of cell senescence and inflammation. These findings suggest that the decreased production of estrogen due to postmenopausal condition altered the osteal macrophage subsets, resulting in a shift toward cell senescence and inflammatory conditions in the bone microenvironment. Furthermore, adoptive macrophage transfer onto calvarial bone was performed, and mice that received oxidatively stressed macrophages exhibited greater osteolytic lesions than control macrophages, suggesting the role of these cells in the development of inflammaging in the bone microenvironment. Consistently, depletion of senescent cells and the oxidatively stressed macrophage subset alleviated the excessive bone loss in postmenopausal mice. Our data provided insight into the pathogenesis of osteoporosis and shed light on a therapeutic approach for the treatment or prevention of postmenopausal osteoporosis.

## Abstract 2

Transcriptome analysis of osteal macrophages demonstrates a shift toward senescent subset in postmenopause, and targeting these cells is beneficial for alleviating bone loss in osteoporosis

## Graphical abstract

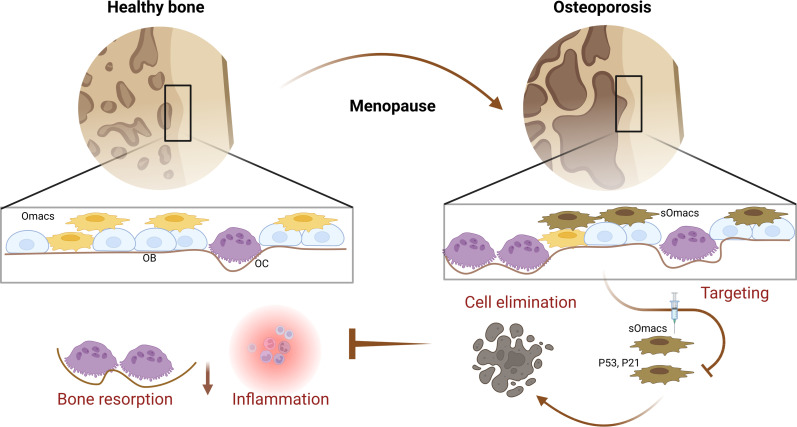

## Introduction

Osteoporosis, a common bone metabolic disease, is characterized by compromised bone volume, decreased mineral density, and decreased strength, resulting in an increased risk of fractures. It is estimated that over 370 million people worldwide were affected by osteoporosis in 2021, and 40% of women and 13% of men of ages of over 50 years are at risk of experiencing 1 or more osteoporotic fractures in their lifetime (1). It is likely that osteoporosis will continue to grow exponentially as the elderly population increases and represents a major health problem worldwide with substantial morbidity and mortality (1–3).

Bone is a metabolically active organ that undergoes continuous remodeling that involves bone forming and resorbing to maintain its architecture and shape. This process is tightly regulated and is dependent on the interplay between the cells in the bone microenvironment, including osteoblasts and osteocytes of mesenchymal origin and osteoclasts and osteal macrophages of hematopoietic origin (4). While osteoblasts and osteoclasts are the key effector participants in bone remodeling, osteocytes and osteal macrophages function as regulatory cells, thus maintaining a homeostatic state in the bone microenvironment (4). Menopause is a major risk factor for skewing this balanced process toward the resorption of excess levels of bone. In fact, women lose up to 10% of their bone mass in the first 5 years after menopause because of a decreased estrogen output, and, as a result, they become highly susceptible to fractures (5). The current pharmacotherapies mainly aim at restoring estrogen levels or a homeostatic state in bone via inhibiting the RANK ligand (RANKL) signaling, which mediates bone resorption or activates Wnt signaling that promotes bone formation. However, there are some health concerns regarding the long-term use of bone resorption inhibitors such as a monoclonal antibody against RANKL and bisphosphonates and bone formation stimulants such as teriparatide and sclerostin (3, 6). Moreover, although estrogen supplementation prevents bone loss and reduces the risk of fracture, it increases the risk of heart attacks, strokes, blood clots, and breast cancer. These collective concerns emphasize the need for alternative therapeutic approaches to the treatment or prevention of osteoporosis (6).

Given the vital functions of macrophages in tissue homeostasis, in addition to their ability to rapidly adapt and respond to environmental cues by secreting osteoactive factors, it has been suggested that osteal macrophages are a major player in bone homeostasis and represent an exciting research area for discovering new therapeutics for bone osteolytic diseases. Osteal macrophages account for about one-sixth of the total cells in bone tissue and are positioned immediately adjacent to resting endosteal and periosteal bone surfaces within bone lining cells. They are morphologically and functionally distinct from osteoclasts and can be distinguished by cell markers that include F4/80, CD68, and Mac3 (7). There are several lines of evidence to suggest that osteal macrophages have essential anabolic functions in bone via promoting osteoblast differentiation and the mineralization required for intramembranous bone healing at fracture sites (8, 9). More recent findings highlighted that osteal macrophages support not only the physiological function of osteoblasts but also osteoclasts via phagocytizing resorption byproducts (10), and their function appears to be essential for maintaining healthy bone mass (11). However, these findings raise the question of whether menopause can alter the anabolic function of osteal macrophages leading to osteoporosis. Since a better understanding of the molecular and functional alterations in osteal macrophages in the postmenopause state should provide a clue for the discovery of therapeutics for the prevention of osteoporosis, the aim of this study was to explore the potential contribution of osteal macrophages to the pathogenesis of osteoporosis using an ovariectomized mouse model. Our results suggest that postmenopausal osteoporosis is associated with an increase in the number of senescent osteal macrophages in the bone microenvironment, and targeting these cells represents a promising approach for developing therapeutics for treatment or prevention of disease progression.

## Results

### Gene profiling of osteal macrophages demonstrates an increase in cellular stress and senescence in an ovariectomy-induced osteoporosis model.

**Figure 1:**
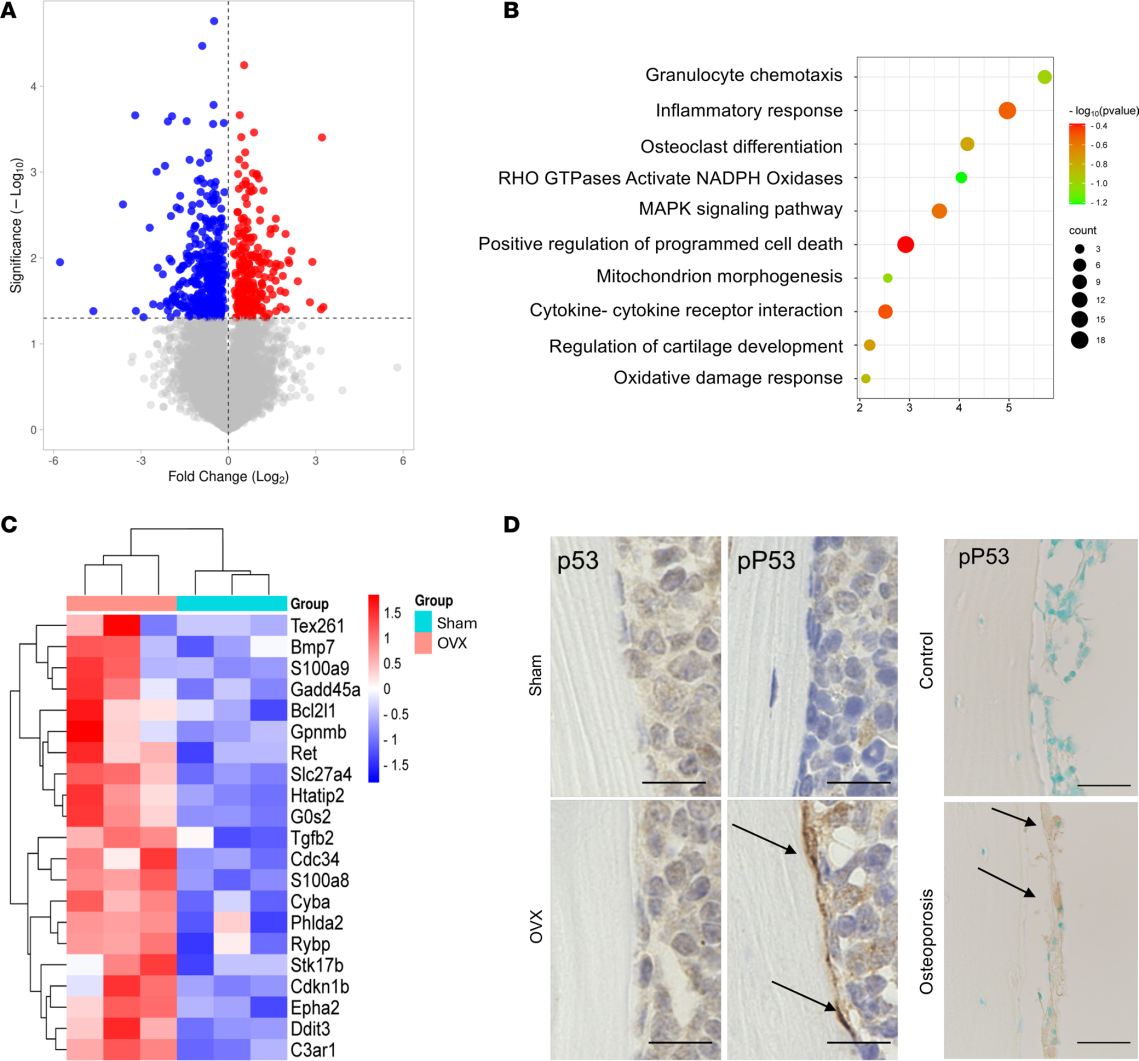
Gene profiling of Omacs in an osteoporosis mouse model assayed by bulk RNA-Seq. (**A**) Volcano plot for gene profile of Omac data from OVX mice as compared with that of sham-treated mice. Red plots represent upregulated genes, blue plots represent downregulated genes (*P* < 0.05), and gray plots represent genes that are not significantly regulated. (**B**) Gene enrichment analysis of genes that are significantly upregulated in OVX-Omacs. (**C**) Heatmap for the expression of genes involved in oxidative stress damage and programmed cell death. (**D**) Immunostaining of femoral bone tissues by P53 and p-P53 in experimental mouse osteoporosis and clinical samples. The left panels represent tissue from experimental mouse osteoporosis, and right panels are from clinical samples. Scale bars are 100 μm. Arrows indicate positive signals of the targets.

To assess the effect of the postmenopausal state on the gene profile of resident osteal macrophages (Omacs), bone lining CD11b^+^F4/80^+^ and C11b^+^CD68^+^ cells from femoral and vertebral bones were isolated from ovariectomized (OVX) mice, designated as OVX-Omacs, and subjected to bulk RNA-Seq analysis. A total of 231 genes were found to be differentially upregulated, and 383 genes were found to be downregulated (FDR < 0.05) in OVX-Omacs (Figure 1A). The top upregulated genes (200 genes) in OVX-Omacs were mostly plotted in downregulated genes of the profiles of other tissue-resident macrophages (ImmGen Consortium) (Supplemental Figure 1A; supplemental material available online with this article; https://doi.org/10.1172/jci.insight.182418DS1). ACP5 (TRAP), a major marker of osteoclasts, was not found in the list of the regulated genes, which suggests the success of specific isolation of Omacs. Moreover, a number of the upregulated genes in OVX-Omacs were found in the gene signature of inflammatory M1 macrophages (Supplemental Figure 1B). To further exploit the molecular and biological characteristics of the gene profile of OVX-Omacs, gene ontology and pathway enrichment analyses were applied to the upregulated genes (Reactome, Kyoto Encyclopedia of Genes and Genomes, FDR < 0.05, fold-change [FC] > 1). The upregulated genes were most significantly enriched (FDR < 0.05) in the positive regulation of chemotaxis, inflammatory response, apoptotic process, and oxidative damage responses for biological process terms (Figure 1B). Pathway database mapping and transcription factor enrichment analyses demonstrated that the top enriched pathway included RHO GTPases Activate NADPH oxidases and MAPK signaling pathway (Figure 1B). Moreover, the transcriptional regulatory network analysis showed that transformation related protein 53 (Trp53) was one of the most significantly enriched transcriptional factors (*P* < 0.05). The increased expression of genes involved in oxidative stress and apoptotic process as well as the activation of Trp53 signaling (Bcl2l1, Cdkn1b, Epha2, Gadd45a, Gpnmb) indicate the development of cellular senescence in OVX-Omacs (Figure 1C and Supplemental Figure 1C). Furthermore, stronger signals representing phosphorylated P53 (p-P53) were detected in the lining cells of OVX mouse femoral bone tissue and sectioned bone of patient osteoporotic femoral bone than were detected in the corresponding tissue of controls (Figure 1D and Supplemental Figure 2A). It is noteworthy that the majority of p-P53–positive cells were the lining cells, and only a small number of p-P53–stained osteocytes were observed in femoral bone tissue of OVX mice (Supplemental Figure 2B). Considering these findings, we conclude that the postmenopausal condition shifts the gene profile of Omacs toward senescence that may lead to the accumulation of senescent myeloid cells in the bone microenvironment.

### Single-cell RNA-Seq identifies a major subset of OVX-Omacs associated with cellular senescence and inflammation.

**Figure 2:**
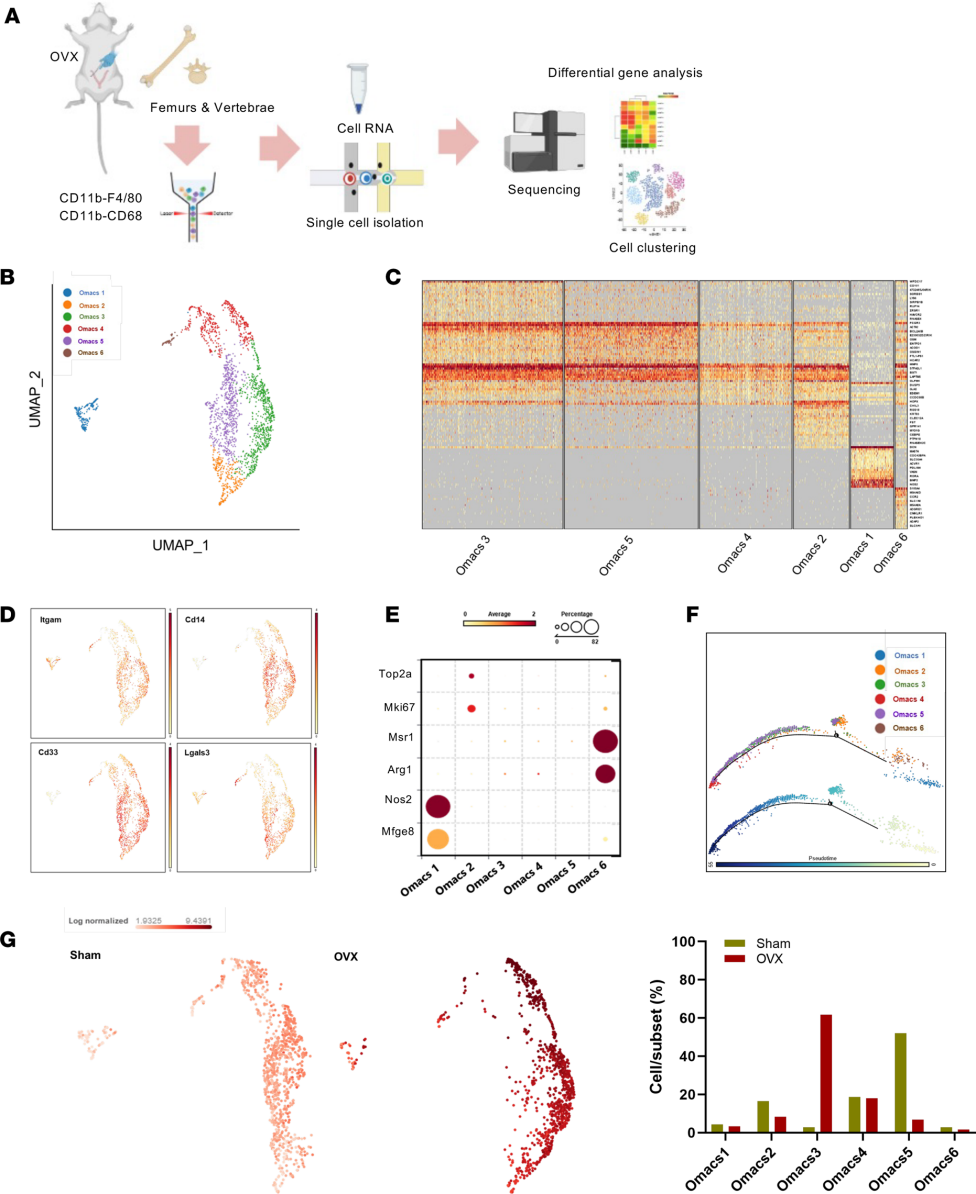
Transcriptional heterogeneity of Omacs and changes in subsets in postmenopausal osteoporosis. (**A**) Schematic outlining the scRNA-Seq analysis. (**B**) Uniform manifold approximation and projection (UMAP) plot of the scRNA-Seq data of Omacs from OVX and sham mice. (**C**) Heatmap for the regulated genes in each cluster. (**D**) Expression of common tissue macrophage markers in the major subsets of Omacs. (**E**) Bubble heatmap for the gene expression of efferocytosis, proliferation, and M2 macrophage markers in subdominant subsets of Omacs. (**F**) Trajectory analysis of the Omacs population. (**G**) Alteration of Omacs population in OVX mice. The right panel shows the relative cell percentage in OVX and sham mice in each cluster.

**Figure 3:**
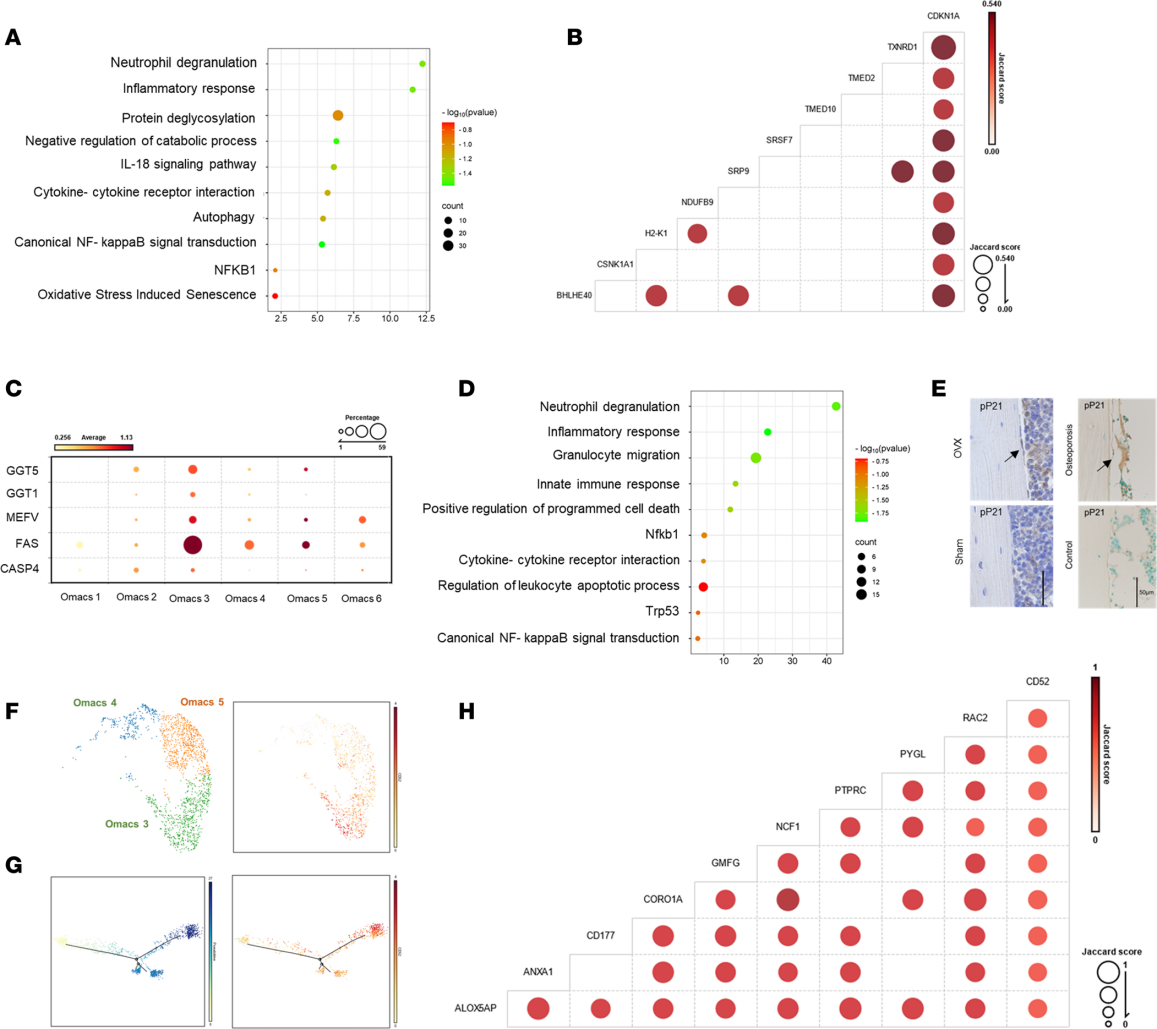
Identification of a major subset of Omacs associated with postmenopausal osteoporosis. (**A**) Gene enrichment analysis of genes that are significantly upregulated in OVX-Omacs based on scRNA-Seq data. (**B**) Jaccard coefficient analysis for the gene set coexpressed in Cdkn1A-expressing cells. (**C**) Bubble heatmap for the gene expression of apoptosis and oxidative stress in each cluster. (**D**) Gene enrichment analysis of genes that are significantly upregulated in Omacs as compared with other populations. Expression of senescence-associated secretory phenotypes (SASPs) in each cluster of Omacs. (**E**) Immunostaining of femoral bone tissues by p-P21 in experimental mouse osteoporosis and clinical samples. Scale bars are 50 μm. Arrows indicate positive cells. (**F**) UMAP plot of color-coded cell cluster of the major Omacs in the bone microenvironment. (**G**) Cluster-specific relative expression of CD52 shown in pseudotime analysis. (**H**) Jaccard coefficient analysis for the top gene set coexpressed in CD52-expressing cells.

To gain a deeper understanding of the changes in the biological and molecular properties of Omacs in osteoporosis, Omacs were collected from OVX and sham mice and subjected to a single-cell RNA-Seq (scRNA-Seq) analysis (Figure 2A). The scRNA-Seq analysis revealed that the Omacs of control and OVX mice were clustered into 6 subsets that were expressing common tissue macrophage markers such as Itgam, Cd33, Cd14, Lgals3, Fcgr3A, and Tlr2 but not bone marrow macrophage markers such as Acp5 and Siglec1 or eosinophil markers such as SiglecF, Itga4, Ccr3, Il5A, and Cx3cr1 (Figure 2, B–D, and Supplemental Figure 3). While the predominant subsets, including Omacs3, Omacs4, and Omacs5, expressed common gene markers of activated myeloid cells, the subdominant subsets, including Omacs1, Omacs2, and Omacs6, expressed unique gene markers that were specific to efferocytosis (Omacs1: Mfge8 & Nos2), proliferation (Omacs2: Mki67 & Top2a), and M2 macrophages (Omacs6: Arg1 & Msr1) (Figure 2, D and E). Consistent with these results, a trajectory analysis of the Omacs population indicated 3 separate directional branches, where Omacs1, 2, 6 mainly formed the focal center and were enriched in the early stage of pseudotime, and Omacs3,4,5 formed side branches and were enriched in the late stage of pseudotime (Figure 2F). More importantly, the OVX mice displayed an altered composition of Omacs that was characterized by a dramatic increase in subset 3 (Omacs3) by approximately 20-fold (Figure 2G). These results demonstrate the transcriptional heterogeneity of Omacs and that the postmenopause period influences and alters their populations in the bone microenvironment. Furthermore, the gene ontology of significantly upregulated genes in OVX-Omacs (81 genes; FC > 0.5 and FDR < 0.05) revealed that these genes were most significantly enriched in inflammatory response, autophagy, and positive regulation of canonical NF-κB signal transduction for biological processes (Figure 3A). The top enriched pathway term included neutrophil degranulation and oxidative stress induced senescence (Figure 3A and Supplemental Figure 4). The Jaccard analysis demonstrated that cells expressing Txnrd1 and Ndufb9, which are associated with increased oxidative stress, are highly expressing Cdkn1a (Figure 3B). Importantly, the expressions of genes associated with increased oxidative stress in macrophages such as Ggt1, Ggt5, Casp4, Fas, and Mefv were significantly elevated in Omacs3 as compared with other OVX-Omacs subsets (Figure 3C). Consistent with these results, the differentially upregulated genes in Omacs3 (FDR < 0.05) as compared with other Omacs were enriched in inflammatory response, positive regulation of programmed cell death, and regulation of canonical NF-κB signal transduction for biological processes and Trp53 for transcriptional regulatory network analysis (Figure 3D). In a comparison with public databases, the majority of differentially upregulated genes in Omacs3 were found to be predominantly expressed in murine senescent cluster cells (SenNET database) and aged myeloid cells (National Center for Biotechnology [NCBI] Gene Expression Omnibus [GEO] GSE145562) collected from liver, lung, peritoneal cells, and spleen (Supplemental Figure 5, A and B). The common genes that were expressed in Omacs3 and aged myeloid cells were most significantly enriched in inflammatory response, positive regulation of programmed cell death, and activate NADPH oxidases transduction for biological processes and Nfkb1 and Trp53 for transcriptional regulatory network analysis (Supplemental Figure 5C). To verify our findings, IHC for detection of p-P21 was performed, and the results showed that strong signals representing p-P21 were detected in the lining cells of femoral bone tissue and sectioned bone of patient osteoporotic femoral bone (Figure 3E). Furthermore, the major subsets including Omacs3, 4, and 5 were subjected to Louvain clustering and trajectory analysis for identifying a specific cell marker of Omacs3. The subset analysis underlined Cd52 as a potential cell marker for Omacs3, and the Cd52-expressing cells were present in the side of the separated branch in the late stage of pseudotime, which indicates the development of the subset because of postmenopausal condition (Figure 3, F and G). The coexpression analysis displayed that there was a correlation between expression of Cd52 and oxidative stress markers such as Rac2, Coro1A, and Alox5AP as well as senescence markers (Figure 3H and Supplemental Figure 6, A and B). Based on a public database of gene expression in the cells, Cd52 seems to be specifically expressed by lymphocytes and myeloid cells (Supplemental Figure 6), and this expression was significantly correlated with expression of oxidative stress markers including Rac2, Coro1A, and Alox5AP (Supplemental Figure 6C). To gain additional evidence on the correlation between senescence and CD52 expression, we reanalyzed an available database for myeloid cells of aging mice and found that aged macrophages from murine liver and lung exhibited increased expression of Cd52 (Supplemental Figure 6D). These collective results revealed that the reduced production of estrogen in ovariectomized mice causes a shift in macrophage populations toward predominant senescent macrophages in the bone microenvironment that express a number of SASPs, thereby promoting inflammation and bone loss.

### Ovariectomy is correlated with an increase in oxidative stress–induced senescence of macrophages.

**Figure 4:**
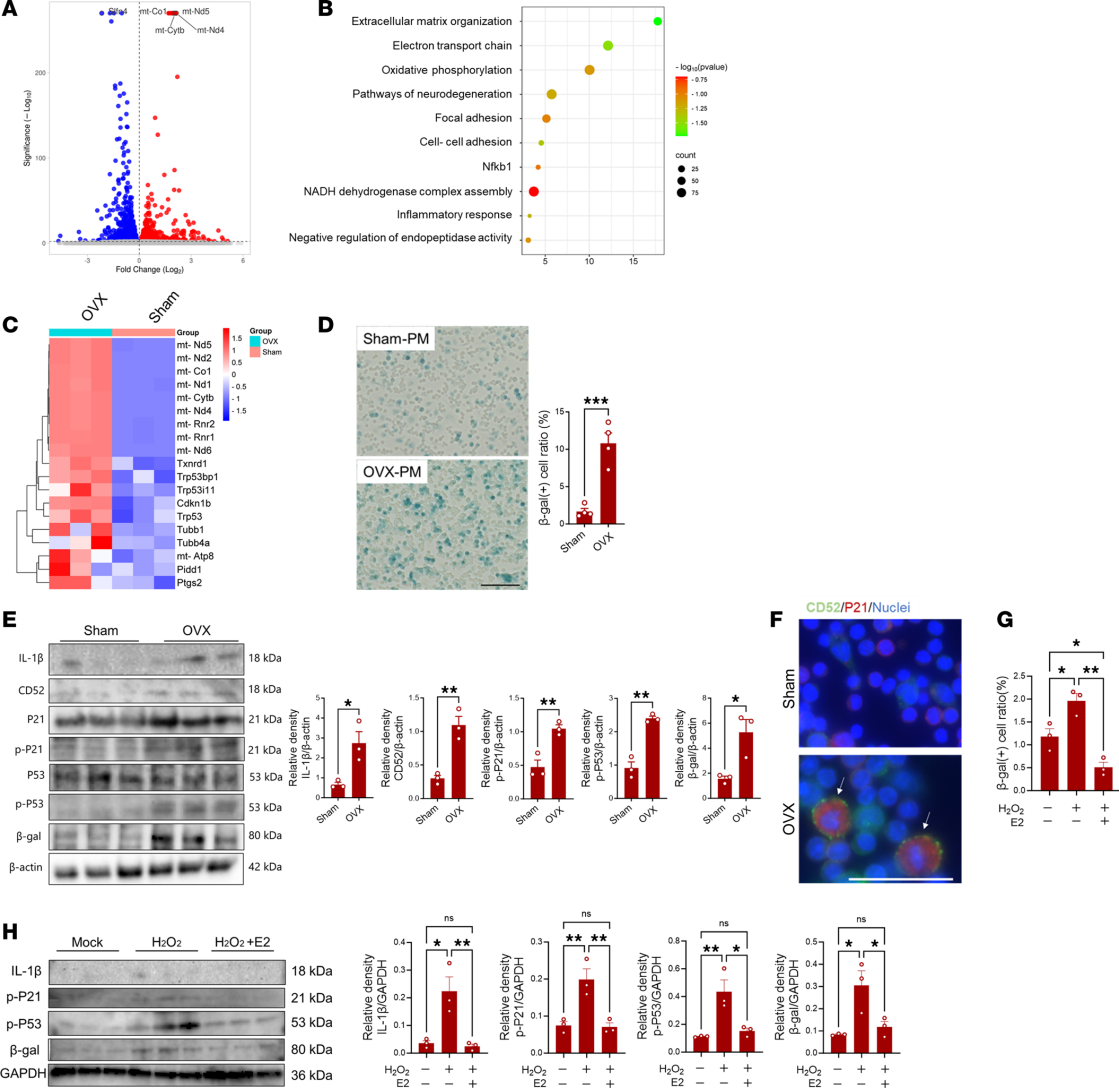
Role of estrogen in development of macrophage senescence. (**A**) Volcano plot for gene profile of peritoneal macrophage data from OVX mice as compared with that of sham mice. Red plots represent upregulated genes, blue plots represent downregulated genes (*P* < 0.05), and gray plots represent genes that are not significantly regulated. (**B**) Gene enrichment analysis of genes that are significantly upregulated in OVX peritoneal macrophages. (**C**) Heatmap for the expression of genes involved in oxidative stress and cellular senescence. (**D**) Percentage of β-gal–positive macrophages collected from peritoneal cavity of sham and OVX mice. The left panel shows representative images for the stained cells, and the right panel shows the percentage of positive cells. (**E**) Western blot analysis of senescence markers in peritoneal macrophages of sham and OVX mice. The right panels show quantification of band density for each marker as assessed by Western blotting. (**F**) Expression of CD52 and P21 in peritoneal macrophages by immunofluorescence test: green for CD52, red for P21, and blue for nuclei. Scale bar is 50 μm. (**G** and **H**) In vitro oxidative stress model (exposure to H_2_O_2_) and treatment by E2. (**G**) Comparison of percentage of β-gal–positive macrophages after exposure to H_2_O_2_ and treatment with E2. (**H**) Western blot analysis for senescence markers in macrophages after exposure to H_2_O_2_. The right panels show the quantification of band density for each marker as assessed by Western blotting. Bars are the mean ± SEM. The significant difference was determined by the 1-way ANOVA, followed by Tukey’s multiple-comparison procedure for multiple-group comparison, and 2-tailed Student’s *t* test for 2-group comparison. * = *P* < 0.05; ** = *P* < 0.01; *** = *P* < 0.001.

To gain direct evidence concerning the correlation between the reduced production of estrogen and the development of macrophage senescence, peritoneal macrophages were collected from the OVX mice and control mice for a gene and protein expression analysis. Bulk RNA-Seq analysis demonstrated a total of 1,223 genes were found to be differentially upregulated and 1,447 genes were found to be downregulated (FDR < 0.05) in peritoneal macrophages of OVX mice as compared with those of sham mice (Figure 4A). Gene ontology and pathway enrichment analyses of the upregulated genes (FDR < 0.05, FC > 1) revealed that the upregulated genes (220 genes) were most significantly enriched (FDR < 0.05) in the oxidative phosphorylation pathway, NADH dehydrogenase pathway, and pathways in neurodegeneration, which are linked to increased oxidative damage and mitochondrial dysfunction (Figure 4B). Moreover, these cells exhibited a remarkable increase in the expression of genes involved in oxidative stress and P53 signaling such as mT-Atp6, mT-Ndu, mT-Co1, Txnrd1, Trp53i11 (Figure 4C). These results were consistent with an elevation in the expression of senescence markers in peritoneal macrophages from OVX mice, as compared with those in sham mice (Figure 4, D and E, and Supplemental Figure 7). Likewise, there was an increase in expression of CD52 and the number of CD52^+^p-P21^+^ cells in peritoneal macrophages isolated from OVX mice as compared with those from sham (Figure 4, E and F). These results verify that decreased estrogen level in the postmenopausal condition causes an increase in oxidative stress in peritoneal macrophages, leading to development of cellular senescence in an analogous manner observed in Omacs. To reinforce the protective role of estrogen on macrophages, cells were subjected to in vitro oxidative stress in the presence or absence of estradiol (E2). Given the findings that Omacs and peritoneal macrophages from OVX mice exhibited a gene signature of NADPH oxidase activation, H_2_O_2_-induced NADPH oxidase activation was used as an in vitro oxidative stress model. Of note, supplementation of E2 to cultured macrophages that had been treated with H_2_O_2_ decreased the harmful effects of oxidative stress on cells and suppressed the gene expression of p-P53, β-gal, p-P21, and IL-1β (Figure 4, G and H). The increase in the expression of senescence markers in peritoneal macrophages exposed to H_2_O_2_ was associated with elevated expression of CD52, suggesting the correlation between the expression of CD52 in macrophages and oxidative stress–induced senescence (Supplemental Figure 8). In line with these results, supplementation of E2 to the OVX mice reduced the number of β-gal– and ROS-stained cells in peritoneal macrophages (Supplemental Figure 9). These collective data demonstrate the existence of a direct correlation between decreased estrogen levels and the accumulation of senescent macrophages in tissue, including bone. The accumulation of senescent macrophages in tissue in the postmenopausal condition is most likely to contribute to inflammaging process in the tissue.

### Senescent macrophages contribute to development of inflammation and bone loss.

**Figure 5:**
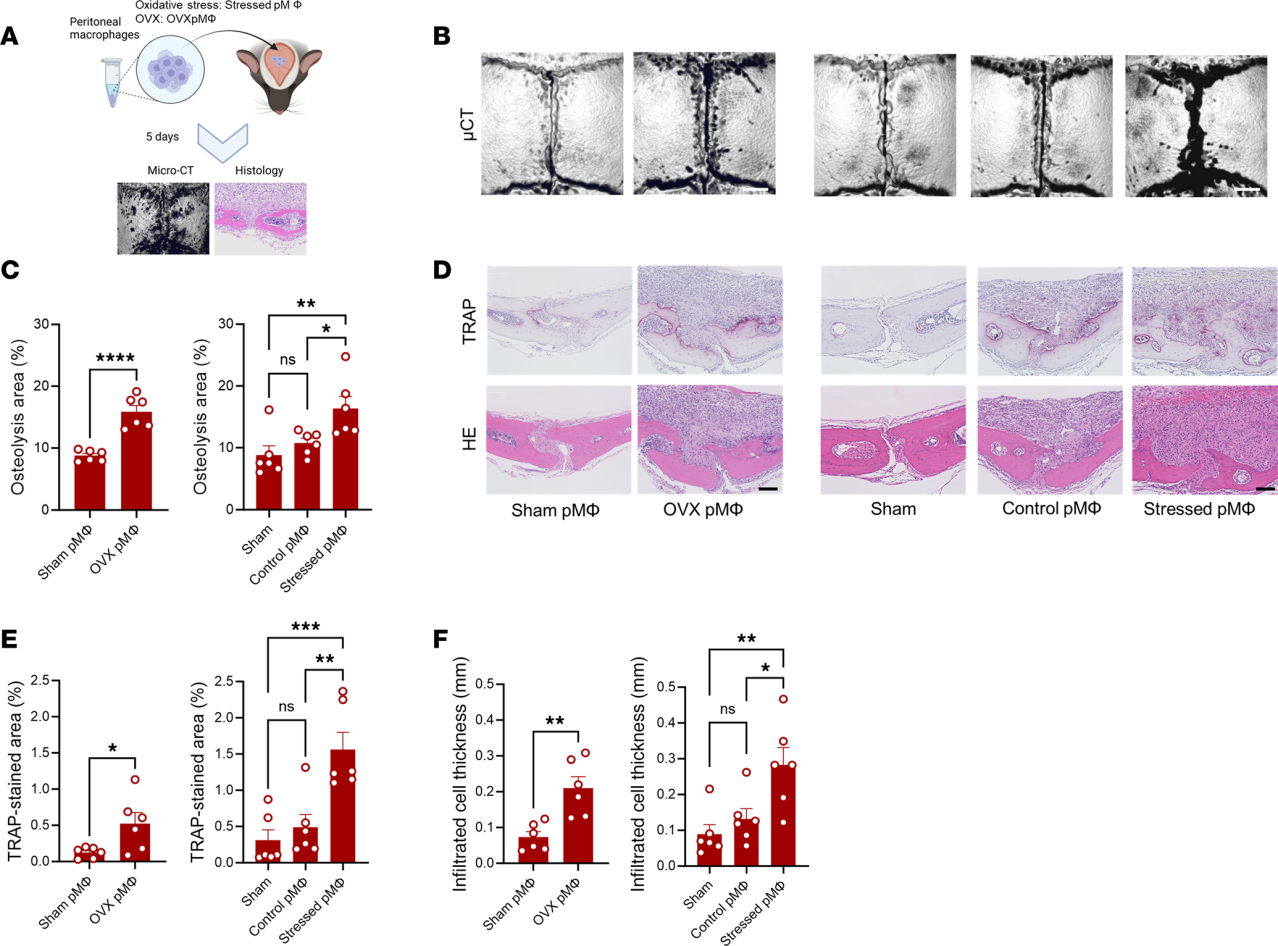
Pathological effects of adoptive transfer of macrophages onto calvarial bone. (**A**) Schematic outlining the experimental design. Peritoneal macrophages from OVX and sham mice as well as from oxidative stress–induced model were adoptively transferred onto calvarial bone for 5 days. (**B**) Representative images for micro-CT analysis. (**C**) Quantification of the lytic area in calvarial bone tissues analyzed by micro-CT. (**D**) Representative images for histological analysis. (**E** and **F**) Quantification of TRAP-stained areas and inflammatory infiltrate in calvarial bone sections. Scale bars are 0.5 mm. Results represent the mean ± SEM of 6 mice. The significant difference was determined by the 1-way ANOVA, followed by Tukey’s multiple-comparison procedure for multiple-group comparison, and 2-tailed Student’s *t* test for 2-group comparison. * = *P* < 0.05; ** = *P* < 0.01; *** = *P* < 0.001; **** = *P* < 0.0001.

To test our supposition that presence or accumulation of senescent macrophages in the bone microenvironment promotes bone loss, peritoneal macrophages from OVX mice and oxidatively stressed macrophages were adoptively transferred onto calvarial bones (Figure 5A). Calvarial bone tissues were harvested after 5 days for histomorphometric and histological analyses. The micro-CT analysis demonstrated that mice that received peritoneal macrophages from OVX mice and oxidatively stressed macrophages developed significantly greater osteolytic lesions compared with the mice that received control macrophages (Figure 5, B and C). Histologically, these mice had larger areas of TRAP staining and inflammation compared with control mice (Figure 5, D–F). These results revealed that senescent macrophages can promote inflammation and bone loss, which emphasizes their role in development of inflammaging in the bone microenvironment in postmenopausal osteoporosis.

### Targeting cellular senescence alleviates bone loss in a postmenopausal osteoporosis model.

**Figure 6:**
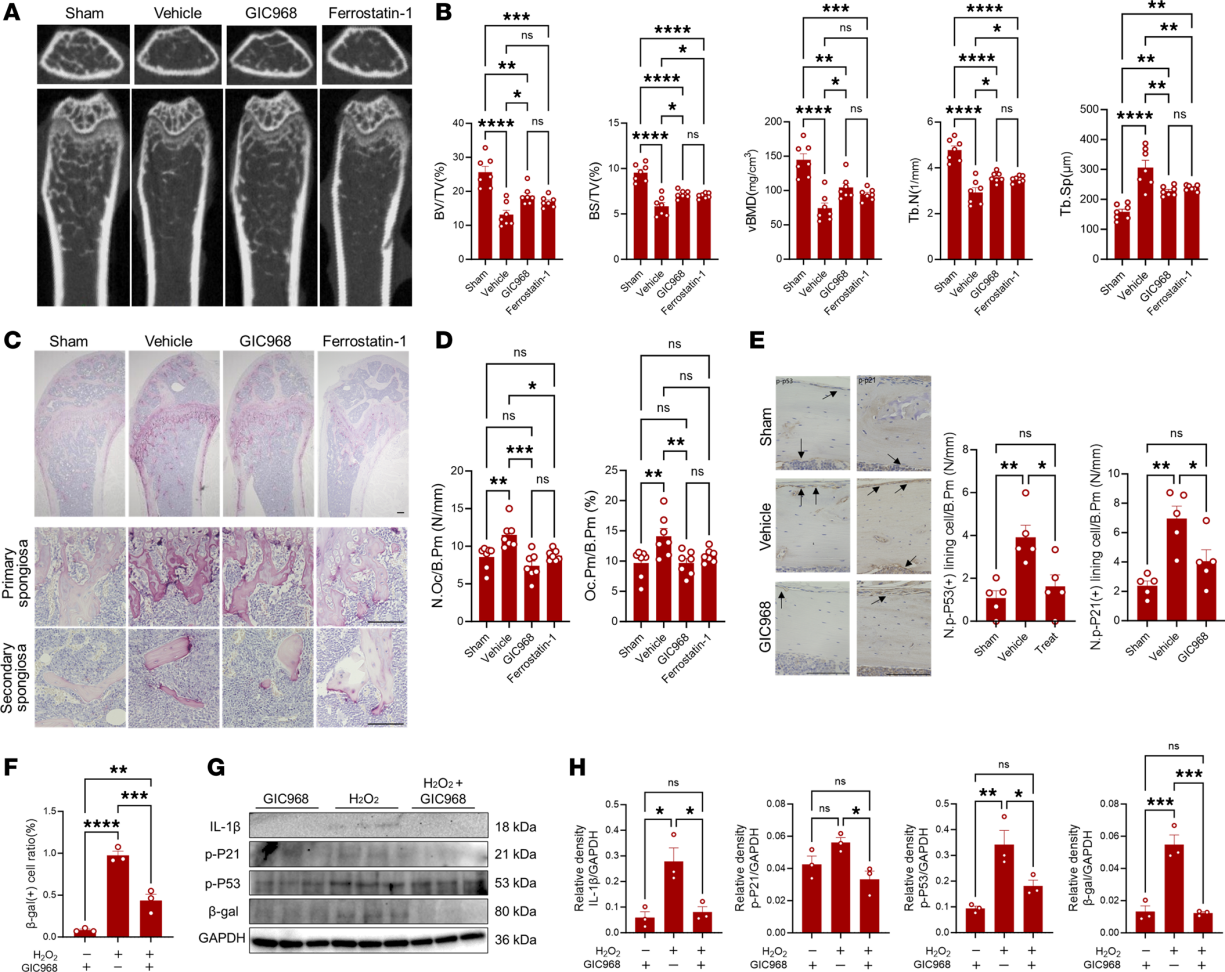
Beneficial effects of using glutaminase inhibitor for elimination of senescent cells in postmenopausal osteoporosis. (**A**) Representative micro-CT images of femoral bones in OVX mice. (**B**) Bone parameters including BV/TV, BS/TV, vBMD, Tb.N, and Tb.Sp of femoral bones. (**C**) TRAP-stained sections of femoral bone. The bottom shows TRAP-stained sections of primary and secondary spongiosa. Scale bars are 100 μm. (**D**) Quantification of osteoclasts’ number and sizes on the surface of femoral bone. Results represent means of 7 samples ± SEM. Significant difference was determined by the 1-way ANOVA, followed by Tukey’s multiple-comparison procedure. * = *P* < 0.05; ** = *P* < 0.01; *** = *P* < 0.001; **** = *P* < 0.0001. (**E**) GIC968 treatment reduced the number of senescent bone lining cells (positive cells for p-P53 and p-P21). Scale bars are 100 μm. Arrows indicate positive singlets of the targets. Right panels show quantification of positively stained cells. (**F**) Comparison of percentage of β-gal–positive macrophages after exposure to H_2_O_2_ and treatment with GIC968. (**G** and **H**) Western blot analysis of senescence markers in macrophages after exposure to H_2_O_2_ and treatment with GIC968. The right panels show the quantification of band density for each marker as assessed by Western blotting. Bars are the mean of 3 samples ± SEM. The significant difference was determined by the 1-way ANOVA followed by Tukey’s multiple-comparison procedure. N.Oc/B.Pm, osteoclast number per bone perimeter; Oc.Pm/B.Pm, osteoclast perimeter per bone perimeter.

**Figure 7:**
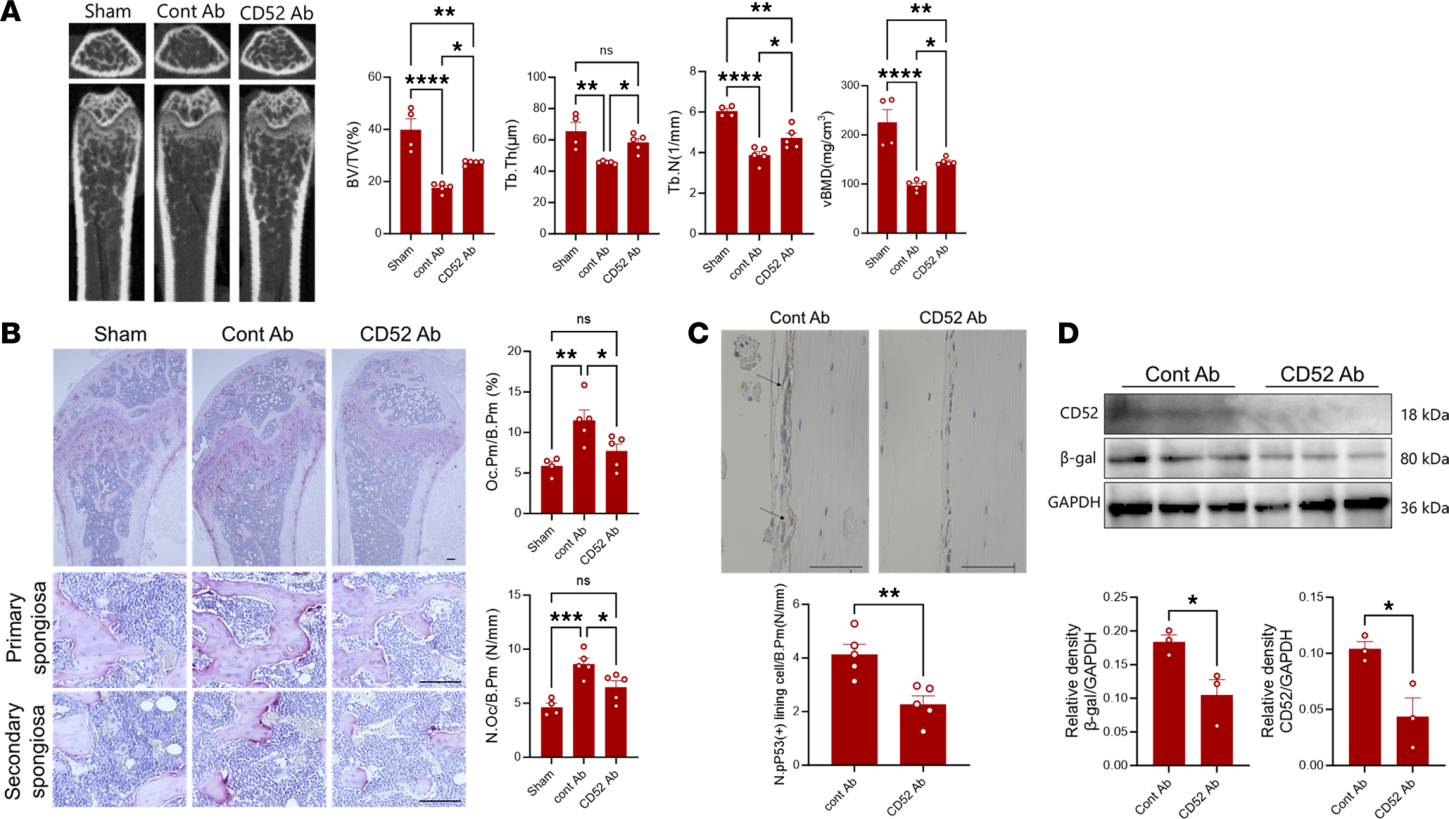
Therapeutic effects of depletion of CD52^+^ cells in OVX SCID mice. (**A**) Representative micro-CT images of femoral bones in OVX mice. The right panels show bone parameters including BV/TV, BS/TV, vBMD, Tb.N, and Tb.Sp of femoral bones. (**B**) TRAP-stained sections of femoral bone, including primary and secondary spongiosa. Scale bars are 100 μm. The right panels show quantification of osteoclasts’ number and sizes on the surface of femoral bone. Results represent means of 5 samples ± SEM. Significant difference was determined by the 2-tailed Student’s *t* test. * = *P* < 0.05; ** = *P* < 0.01; *** = *P* < 0.001; **** = *P* < 0.0001. (**C**) Detection of p-P53–positive lining cells in femoral bone tissues by IHC. The upper panel represents IHC sections stained with antibody against p-P53 in femoral bone tissue. The lower panel is a quantification of positive cells. Results represent cell count of 5 samples ± SEM. Significant difference was determined by the 2-tailed Student’s *t* test. Scale bars are 50 μm. Arrows indicate positive signals of the p-P53. (**D**) Western blot analysis for senescence markers in peritoneal macrophages. The lower panels show the quantification of band density for each target. Results represent means of 3 samples ± SEM. Significant difference was determined by the 2-tailed Student’s *t* test.

Given the findings that the accumulation of senescent Omacs in the bone microenvironment is associated with initiation of inflammation and bone loss, we further examined the potential beneficial effects of targeting these cells in the postmenopausal osteoporosis model. Glutaminase inhibitor compound (GIC) 968 that inhibits mitochondrial glutaminolysis, resulting in a rapid and specific elimination of senescent cells, and ferrostatin-1 that suppresses the cellular increase of oxidative stress, leading to survival of the cells, were used for the treatment of postmenopausal osteoporosis. The inhibitors were intraperitoneally administered 3 times per week 1 week after ovariectomy for 3 weeks, and femoral and vertebral bones were then harvested for histomorphometric and histological analyses. Importantly, a micro-CT analysis demonstrated that GIC968 treatment alleviated the bone loss caused by OVX in mouse femurs, as evidenced by the higher levels of bone volume per tissue volume (BV/TV), bone surface/tissue volume (BS/TV), volumetric bone mineral density (vBMD), and trabecular number (Tb.N), and a lesser increase in trabecular separation (Tb.Sp) in these mice as compared with the corresponding values for the vehicle-treated (control) OVX mice (Figure 6, A and B). Ferrostatin-1 treatment slightly improved these bone parameters, but there were no statistically significant differences with control OVX mice in terms of BV/TV and vBMD parameters (Figure 6, A and B). Consistent with these observations, the histological analysis revealed that the GIC968-treated OVX mice exhibited a significant reduction in the numbers of TRAP-positive osteoclasts in the secondary spongiosa of femurs (Figure 6, C and D). Given the findings that GIC968 treatment reduced the number of p-P53– and p-P21–positive lining cells in femoral bone tissue (Figure 6E), we next verified the beneficial effects of GIC968 in eliminating senescent macrophages after exposure to oxidative stress in vitro. It is noteworthy that the GIC968 treatment reduced the expression of senescence markers in stressed macrophages (Figure 6, F–H). This treatment seemed to have no effects on differentiated osteoclasts in vitro, as it did not significantly reduce the number of TRAP^+^ cells (Supplemental Figure 10). These results reinforce the findings that targeting oxidative stress–induced senescent macrophages might be beneficial to reduce pathological bone resorption in early-stage postmenopausal osteoporosis. Consistent with these results, oral administration of senolytic drugs dasatinib (D) and quercetin (Q) ameliorated the excessive bone loss in OVX mice and reduced the number of TRAP-positive osteoclasts in femur tissues and number of β-gal–positive peritoneal macrophages (Supplemental Figure 11, A–C). Likewise, the number of β-gal–positive macrophages tended to be reduced in oxidative stress–exposed macrophages that were treated with D+Q in vitro (Supplemental Figure 11D). Furthermore, an attempt to reduce the accumulation of senescent macrophages in the tissue was carried out by using a specific antibody against CD52 to deplete Omacs3 in SCID mice after ovariectomy. Depletion of CD52^+^ cells by specific antibody treatment alleviated the bone loss in OVX mouse femurs, as evidenced by the higher levels of BV/TV and vBMD, accompanied with a significant reduction in the numbers of TRAP-positive osteoclasts in the secondary spongiosa of femurs in recipient OVX mice as compared with control (Figure 7, A and B). Moreover, this treatment resulted in a significant reduction in the number of p-P53–positive lining cells and the expression of senescence markers in the peritoneal macrophages as compared with those of the control (Figure 7, C and D). It is worth mentioning that bone parameters tended to be increased in the vertebral bones of GIC968-, senolytic drug–, and CD52 antibody–treated OVX mice as compared with control mice (Supplemental Figure 12). These results suggest that reducing the number of senescent Omacs in the bone microenvironment is beneficial for suppressing excessive bone loss in postmenopausal osteoporosis. These collective findings suggest that targeting senescent Omacs represents a promising therapeutic approach for the treatment or prevention of postmenopausal osteoporosis.

## Discussion

Postmenopausal osteoporosis is typified by 2 phases of bone loss, the first of which occurs at the start of menopause and is characterized by an increase in bone resorption in trabecular bone, a process that is referred to as menopause-related bone loss. The later phase occurs 4–8 years later and is characterized by a persistent, slower loss in trabecular and cortical bone due to reduced bone formation. This is essentially the result of decreased estrogen output that is accompanied by an increase in factors promoting bone resorption (12, 13). Given the fact that macrophages have homeostatic functions in tissues and are able to have osteoactive characteristics in response to environmental cues, we explored the molecular changes that occur in Omacs during postmenopausal osteoporosis toward development of a therapeutic approach.

Our study demonstrated the transcriptional heterogeneity of Omacs and identified a subset that is associated with postmenopausal osteoporosis. Postmenopausal condition led to an alteration of Omac subsets shifting toward a predominant cell subset with a typical gene signature of cell senescence and inflammation. Osteal and peritoneal macrophages collected from postmenopausal osteoporosis mice exhibited a gene signature of NADPH oxidase activation, which is known as a key modulator in aging and cell senescence. This appears to be consistent with findings highlighting the fact that a shortage of estrogen is associated with an increased production of senescent cells in estrogen-responsive organs, thereby promoting the onset and progression of age-related diseases, such as pulmonary dysfunction, cardiovascular disorders, neurodegeneration, and diabetes (14–16). At the cellular level, estrogen appears to alleviate senescence via regulating different pathways, including telomerase activity in epithelial cells, the ERβ/SATB2 pathway in bone marrow stromal cells, and the JAK2/STAT3 pathway in bone marrow mesenchymal stem cells (16–18). In fact, it is well established that estrogen acts as a physiological activating signal in macrophages, regulating their ontogenesis, self-renewal, function specialization, and lineage heterogeneity (15). Estrogen modulates the phagocytic activity and polarization of macrophages, shifting their population toward antiinflammatory and reparative phenotypes (15, 19, 20).

Consistent with the findings demonstrating a link between estrogen and the reduced macrophage efferocytosis, our results showed that OVX mice exhibited a decreased number of efferocytic Omacs (Arg1-, Mertk-, Mfge8-expressing Omacs), which are essential for the maintenance and restoration of tissue homeostasis (21, 22). Efferocytosis is a fundamental physiologic process that is associated with phagocytic cells and mediates the clearance of dead cells from tissue and the resolution of inflammation and tissue repair (23). A deficiency of efferocytosis has been frequently reported in aging and chronic inflammatory diseases, as typified by the accumulation of dead cells that lead to autoimmunity, pathological inflammation, and tissue necrosis (23). These findings may explain the sustained bone loss in osteolytic bone diseases, since a lack of efferocytic macrophages would be expected to lead to the accumulation of dead cells and degraded bone by-products at the site of bone resorption, thereby promoting inflammatory bone loss (4, 10, 11). Taken together, these findings suggest that the postmenopausal condition causes an accumulation of senescent Omacs, of which their appropriate and homeostatic functions are impaired in the bone microenvironment, in addition to their ability to produce an array of SASPs that mediate sustained bone loss.

Our results are consistent with earlier findings highlighting the fact that the accumulation of senescent myeloid cells in a bone microenvironment contributes to the development of bone loss in older people (24, 25). Moreover, there is evidence to suggest that p16-induced cellular senescence in macrophages skews these cells toward the inflammatory M1 phenotype that impairs bone metabolism and mediates skeletal fragility. In a related study, Li and colleagues reported that p16-deficient mice exhibit a reduction in bone cell senescence and oxidative stress that eventually led to increases in bone mineral density, trabecular bone volume, and the numbers of osteoblasts with a total collagen-positive area in OVX mice (26). Cellular senescence plays a key role in the development of inflammation because of an increase in SASPs consisting of proinflammatory cytokines, chemokines, and extracellular matrix–degrading enzymes that exert deleterious effects on tissue homeostasis.

Cellular senescence is generally thought to be a state in which a cell responds to numerous stressors, such as nutrient deprivation, hypoxia, mitochondrial dysfunction, and genotoxic agents that trigger the activation of the p16/Rb and p21/p53 pathways. The major phenotypical features of senescent cells include irreversible cell cycle arrest and resistance to apoptosis with increased secretory features. Macromolecular damage, proteotoxicity, and the accumulation of misfolded proteins are hallmark events that occur in senescent cells and help identify the process of senescence (24, 27). It should be noted here that these results demonstrate that OVX-Omacs exhibited a gene signature of proteolysis dysregulation that is associated with the accumulation of misfolded and damaged proteins in cells. Protein degradation is a key physiological process that is essential for maintaining cell functions, and the accumulation of misfolded and damaged proteins in cells can have potentially harmful consequences to the cells, which increases the risk of chronic inflammation and the development of age-related diseases (28). This can be explained based on the fact that estrogen is involved in the regulation of proteolysis of ER proteins via a proteasome-mediated pathway (29). Therefore, the issue of how estrogen shortage causes proteolysis dysregulation–mediated senescence in Omacs represents a subject of further study. Senescence and secretion of SASPs are known to drive inflammaging, a process typified by the development of low-grade inflammation in the tissue of aging populations (30). Inflammaging creates a vicious cycle of inflammation and senescence accompanied by development of cellular immunosenescence, organ dysfunction, tissue damage, and age-related diseases. In fact, inflammaging is the driving force in age-related bone loss (31) and represents a promising target for treatment (32). Aged tissue macrophages display a significant upregulation of cell cycle checkpoint inhibitors p16INK4a and p21CIP1, dysfunctional mitochondria, and deficiency in repairing DNA damage accompanied by a noteworthy increase in transcription factors regulating the production of SASP, such as TNF-α, IL-6, MMP9, and IL-1β (33). In this process, oxidative damage due to ROS increase acts as positive feedback loops that drive the accumulation of senescent cells and inflammation. Importantly, supplementation of estrogen in OVX mice reduced cellular ROS increase and expression of senescence markers in peritoneal macrophages. These collective findings suggest that the shortage of estrogen in menopause exacerbates accumulation of senescent Omacs that may contribute to development of inflammaging in the bone microenvironment via producing SASP, thereby promoting excessive bone loss.

Furthermore, the most striking finding in the current study is that the elimination of senescent myeloid cells by systemic administration of GIC968 alleviated excessive bone loss in OVX mice. Although GIC968 targets senescence in all types of cells, not only macrophages, our data and earlier reports clearly showed that myeloid lining cells are the major cells expressing senescence markers in the bone microenvironment in osteoporosis (34). These findings are consistent with earlier findings suggesting that targeting senescence using senolytics offers a transformational strategy for alleviating bone loss and the risk of fractures associated with osteoporosis (34–36). In support of our finding, the elimination of senescent cells by the inhibition of glutaminase 1 has been shown to ameliorate tissue microinflammation and prevent age-associated disorders (37). Senolytics that selectively eliminate senescent cells appear to have beneficial effects on the skeletal system by improving physical function and activities in old age (38). Indeed, there is solid evidence from experimental aging models that senotherapeutics represent a powerful antiaging strategy in that it delays the onset of chronic diseases associated with aging, improves the function of organs, and extends healthspan (39–43). Likewise, depletion of Omacs3 that represents the major senescent myeloid cells in bone using antibody against CD52 suppressed excessive bone loss in OVX SCID mice, which reinforces the importance of targeting senescent myeloid cells in osteoporosis. The antibody against CD52 (CAMPATH-1 antigen, a glycoprotein present on the surface of mature lymphocytes and inflamed monocytes and dendritic cells) has been traditionally used for treatment of B cell chronic lymphocytic leukemia. Antibody against CD52 (alemtuzumab) has shown good effects for treatment of multiple sclerosis and asthma in clinical trials and colitis in an experimental mouse model (44–46). This treatment seemed to be effective as an immunosuppressant for prolonging organ transplantation (47, 48). Essentially, in multiple sclerosis, asthma, and organ/cell transplantation rejection, macrophages and senescence play an important role in the pathogenesis (49–51). Therefore, there is a possibility that the therapeutic effect of alemtuzumab might come from its ability to eliminate senescent myeloid cells. Further study should be directed to address the usefulness of CD52 as a marker for differentiation of senescent tissue macrophages.

In conclusion, our study demonstrates the transcriptional heterogeneity of Omacs and changes in subsets that occur in postmenopausal osteoporosis. The postmenopausal period appears to promote senescence in Omacs that can maintain an inflammatory state in bone tissue, leading to excessive bone loss. Targeting senescence in Omacs offers a promising strategy for preventing postmenopausal osteoporosis.

## Methods

### Sex as a biological variable.

Our study exclusively examined female mice because postmenopausal osteoporosis is only relevant in females. It is unknown whether the findings are relevant for male mice.

### Study design.

The aim of this study was to explore the molecular changes in Omacs in the postmenopausal state and to define a pathogenic cell subset associated with osteoporosis. To this end, we first isolated Omacs using specific antibodies against CD11b, F4/80, and CD68 and then analyzed their molecular changes in OVX mice using bulk and single-cell transcriptomics. Analyses showed that postmenopause alters the Omac subsets, resulting in a shift toward cell senescence and inflammatory conditions in the bone microenvironment. In fact, the postmenopausal mice exhibited a 20-fold increase in Omacs3 with a typical gene signature of cell senescence and inflammation. Subset analysis for cell marker allowed us to identify CD52 as a cell marker of Omacs3. We next performed bulk RNA-Seq analysis on the peritoneal macrophages and found that the postmenopause period alters the peritoneal macrophages in a similar manner to Omacs, as both analyses demonstrated evidence of increased oxidative stress and cell senescence. We further verified the findings in vitro using an oxidative stress model and in vivo osteolytic model using transferred macrophages onto calvarial bone. Furthermore, we examined the usefulness of targeting these cells in vivo in an osteoporosis model using glutaminase inhibitor GIC968, antioxidant ferrostatin-1, D+Q, or CD52 antibody. No prior sample size calculation was performed. All in vitro experiments were repeated at least 2 times for reproducibility of data. Quantitative experiments were evaluated 2 times independently while masked to experiment groups.

### Murine osteoporosis model.

Mice were specific pathogen–free (SPF) mice, maintained under SPF conditions at a controlled temperature of 21°C–22°C, a humidity of 40%–50%, and a constant 12-hour light/12-hour dark room during the experiments. The SPF-BALB/c 10-week-old female mice and C.B17/Icr-scid 10-week-old female mice (CLEA) were anesthetized by an intraperitoneal injection of 100 mg/kg of ketamine and 10 mg/kg of xylazine for ovariectomies (bilateral) (52). Mice were sacrificed 4 weeks after surgery, and their femurs and vertebrae were further subjected to micro-CT analysis and bone histomorphometry analysis.

### Human samples.

Bone tissues were obtained from the operation site of an osteoporosis-diagnosed 60-year-old woman and non-osteoporosis-diagnosed 53-year-old woman. Samples were fixed by paraformaldehyde (Merck), declassified by treatment with EDTA (Wako) for 6 weeks, and embedded in paraffin for further analysis.

### IHC.

Paraffin-embedded sections were incubated at 4°C overnight with the primary antibodies against β-galactosidase (Invitrogen, Thermo Fisher Scientific), p-53 (GeneTex), phospho-p53 (GeneTex), p21 (GeneTex), and phospho-p21 (Bioss) (Supplemental Figure 17), according to the manufacturer’s instructions. Sections were then incubated with a secondary antibody (EnVision+ System-HRP Labeled Polymer; Dako) for 30 minutes. Signals were detected by treating the sections with 3,3-diaminobenzidine, followed by counterstaining with Mayer’s hematoxylin for detecting cellular nuclei (Dako).

### Bone morphometry.

Femurs and vertebrae of mice were harvested and fixed in 10% formalin (Wako) for 24 hours and then scanned by micro-CT (R_mCT2) at a 10 μm isotropic resolution, and x-ray energy was 80 kV and 80 mA. Parameters, such as the BS/BV, BV/TV, Tb.N, Tb.Sp, vBMD, and porosity, were measured using a TRI/3D-BON (Ratoc System Engineering Co.) (52). For bone histomorphometric analyses, fixed femurs were decalcified in EDTA (pH 7.0) for 3 weeks and embedded in paraffin, and 5 μm–thick longitudinal sections were prepared and stained with TRAP (MilliporeSigma) with a methyl green counterstain to observe osteoclasts. The numbers of osteoclasts/bone surface (N.Oc/B●Pm) and size (Oc.Pm/B●Pm) at the secondary spongiosa were determined using the ImageJ software (NIH). The area 250 μm proximal to the growth plate was defined as the primary spongiosa, and that of 250 μm to 1,000 μm proximal to the growth plate was defined as the secondary spongiosa (52).

### Isolation of Omacs.

Femurs and vertebrae of OVX and sham BALB/c mice (20 mice for each condition) were harvested 4 weeks after the ovariectomy. Periosteum was removed for cells’ isolation. For femurs, resection of the epiphysis was performed, and then bone marrow cavity was carefully washed with PBS (2 times) to remove marrow cells using needle and syringe. The procedure was applied to OVX and sham mice in the same manner to avoid any potential anatomical bias. Bone tissues were cut into small pieces and then digested with 0.25% trypsin at 37°C for 30 minutes followed by 0.25 U/mL collagenase D (Roche Diagnostics) at 37°C overnight. The cells were then sorted using the fluorescence-activated cell sorting with specific antibodies against CD11b, F4/80, CD68 (BioLegend) (Supplemental Figures 13 and 17), which are known as Omac markers (7, 10).

### Bulk RNA-Seq analysis.

Isolated Omacs and peritoneal macrophages were lysed with TRIzol Reagent (Invitrogen, Thermo Fisher Scientific), and RNA was extracted using NucleoSpin RNA (TAKARA) according to the manufacturer’s instructions. Libraries were generated from 1 μg of RNA using a NEBNext Ultra II Directional RNA Library Prep Kit (New England Biolabs) and sequenced using Illumina NovaSeq 6000. Data of 150 bp × 2 paired-end, 4 G bases per sample, and 26.7 million reads per sample, were trimmed by fastp, then mapped by alignment to the mouse mm10 reference genome using Hisat2. FeatureCount v1.55.0-p3 was used to count the reads, and fragments per kilobase of transcript sequence per million base pairs sequenced of each gene was calculated based on the length of the gene and read (53). Differential expression analysis was performed using Subio Platform Plug-ins. The *P* values were adjusted using Benjamini-Hochberg approach for the FDR. Differentially expressed genes (DEGs) were determined when FDR < 0.05, and the resulting genes were next subjected to gene ontology enrichment and pathway enrichment analyses.

### scRNA-Seq analysis.

Isolated Omacs were washed once with ice-cold PBS containing 0.04% weight/volume BSA and counted using AO/PI staining by CellDropFL (DeNovix). The cells were then subjected to cell hashing (TotalSeq; BioLegend), and the resulting cells (1,037 for OVX) (1,084 cells for sham) were loaded to Chromium (10x Genomics) and run with Chromium Next GEM Single Cell 3’ Reagent Kits v3.1 through the library preparation procedures following the manufacturers’ instructions. The libraries were quantified using a KAPA Library Quantification Kit (KAPA Biosystems) and sequenced via MGI DNBSEQ-G400 (MGI Tech) (Supplemental Figure 14). A total of 435,107,683 reads were obtained, the raw data from each sample were demultiplexed and aligned to the mm10 reference genome, and unique molecular identifier counts were quantified using the 10x Genomics Cell Ranger pipeline (v6.0.1). The analyses, including clustering and trajectory, and defining DEGs and specific marker of each subset/cluster, were performed by BBrowserX software (BioTuring; https://bioturing.com/single-cell-analysis-bbrowserx). For verification of results of scRNA-Seq, IHC staining of femoral bone tissues was performed for detection of clustered subsets of Omacs using specific antibodies targeting MRC2 (Thermo Fisher Scientific) for Omacs1 and MFGE8 (Thermo Fisher Scientific) for Omacs2 (Supplemental Figures 15 and 17).

### Bioinformatics.

The DEGs were subjected to gene ontology enrichment and pathway enrichment analyses, which were performed based on the databases Metascape (https://metascape.org/gp/index.html#/main/step1) and TRRUST (https://www.grnpedia.org/trrust/). Data visualization and graphing were performed using SRplot tools (http://www.bioinformatics.com.cn/srplot), Venny 2.1, and ImmGen (https://www.immgen.org/Databrowser19/DatabrowserPage.html). The datasets for gene signature of macrophages (GSE69607 and GSE38705) were used to compare the gene profile of Omacs. Aging myeloid cell data (GSE145562) were used to show the expression of CD52 (https://artyomovlab.wustl.edu/sce/?token=DT-1481_Myeloid). BioTuring database for 6,641,383 cells was used to compare the expression of CD52 in different type of cells (https://bioturing.com).

### Osteoclast assay.

Human primary monocytes were isolated from the blood samples of a healthy donor recruited from Hokkaido University Graduate School of Medicine by density gradient centrifugation (Ficoll-Paque PLUS; GE Healthcare, now Cytiva) and differentiated into osteoclasts as described in a previous study (54). Briefly, attached cells were cultured in MEM supplemented with 10% heat-FCS, 5% mg/L penicillin/streptomycin solution, 5% l-glutamine, 25 ng/mL of recombinant macrophage colony-stimulating factor (PeproTech), and recombinant RANKL (sRANKL, PeproTech) in a 37°C humidified atmosphere containing 5% CO_2_ for 6 days. Cultures were replenished with the same fresh medium every 3 days; 10 μM GIC968 (Cayman Chemical) was added to medium on day 6. Cells were stained on day 8 by leukocyte acid phosphate TRAP kit (MilliporeSigma), and cell count was determined as the number of TRAP-positive multinucleated cells/cm^2^ on 5 random microscopic fields in each well.

### E2 treatment model.

The OVX BALB/c mice were randomly divided into 3 groups (4 mice per group) and were intraperitoneally injected with either 5 μg/kg of β-estradiol (MedChemExpress) or the same volume of PBS 5 times/wk over a period of 3 weeks, starting from day 4 after the ovariectomy. Peritoneal macrophages were isolated 1 month after ovariectomy, seeded onto 96-well plates, and cultured in the growth medium of MEM supplemented with 10% heat-inactivated fetal bovine serum (FBS; MilliporeSigma), 1 mM l-glutamine, and 25 mg/L penicillin/streptomycin overnight at 37°C within 5% CO_2_ incubator. Thereafter, macrophages were subjected to a senescence β-galactosidase staining kit (Cell Signaling Technology) for evaluating senescence β-gal activity and ROS Assay Kit-Photo-oxidation Resistant DCFH-DA Kit (DOJINDO) for detecting oxidative stress.

### Procedures for treatment of mice.

The OVX BALB/c mice were randomly divided into 4 groups (7 mice per group) and were intraperitoneally injected with either 10 mg/kg of GIC968 or ferrostatin-1 (Cayman Chemical) in 500 μL 5% DMSO (Wako) or the same volume of vehicle 3 times per week for 3 weeks starting at day 8 after the surgery. Moreover, a senolytic drug including D and Q (Cayman Chemical) was administrated via oral gavage to the OVX BALB/c mice. The senolytic drug containing D (12 mg/kg) and Q (50 mg/kg) was dissolved, diluted in propylene glycol (Wako), and administrated in a volume of 200 μL/mouse on days 7, 8, 9, 21, 22, 23 after ovariectomy. Control mice were treated with vehicle solution following the same procedure. In a separate experiment, OVX C.B17/Icr-scid mice (5 mice per group) were intraperitoneally injected with 10 μg anti-mouse CD52 monoclonal antibody (MBL) for depletion of CD52^+^ macrophages or control isotype antibody (BioXCell) (Supplemental Figure 17) on days 14, 18, 21, 25 after surgery.

### Macrophage transfer onto calvarial bone.

Peritoneal macrophages were isolated from OVX or sham 10-week-old SPF-BALB/c female mice (CLEA) 1 month after ovariectomy. Moreover, peritoneal macrophages were isolated from 8-week-old SPF-BALB/c male mice (CLEA) and then cultured for 60 minutes in the presence or absence of 100 μM H_2_O_2_ (Wako) to initiate oxidative stress damage in macrophages. Thereafter, 8-week-old male SPF-BALB/c mice (6 mice per group) were anesthetized by intraperitoneal injection of 100 mg/kg ketamine and 10 mg/kg xylazine, and sagittal incision (~1 cm) was made over the calvarial anterior site for implantation of macrophages (1 × 10^6^) dissolved in 100 μL PBS. The incision was closed using stainless steel clips. The mice were anesthetized and euthanized by cervical dislocation, and then their calvariae were collected on day 5 after implantation. In a separate experiment, peritoneal macrophages were isolated from OVX or sham 10-week-old SPF-C57BL/6-Tg(CAG-EGFP) female mice (Japan SLC) and grafted onto the calvarial site of 8-week-old male C57BL/6JJcl mice (CLEA) for tracing the implanted macrophages as the cells are expressing green fluorescent protein (GFP). Calvariae were collected on day 1 and 3 after implantation, and presence of the GFP-positive cells was examined by IHC using specific antibody (Supplemental Figure 17). Cells were detected on both days 1 and 3 in both groups (Supplemental Figure 16). Thereafter, micro-CT and bone histomorphometry analysis were conducted as previously reported (54, 55). Briefly, fixed calvariae were analyzed by micro-CT at a 10 mm isotropic resolution. Percentage of bone pits on the calvariae were calculated by ImageJ. The 1 cm^2^ area including the center line of each calvaria was selected for evaluation as the region of interest. For bone histomorphometric analysis, fixed calvariae were decalcified in EDTA (pH 7.0) for 3 days and then embedded in paraffin. The 5 μm–thick sections were stained with hematoxylin and eosin, along with TRAP staining (Wako) according to the manufacturer’s instructions. The 1 cm square, including the center line of each calvaria, was cut and divided into 3 parts, and the 2 distal regions from each stained sections were analyzed by ImageJ for the quantification of cell infiltration into tissues.

### In vitro stress model of macrophages.

Peritoneal macrophages were isolated from 8-week-old SPF-BALB/c mice (CLEA), 3 days after peritoneal injection of 4% brewer thioglycollate medium (BD Biosciences). Cells were washed, seeded onto 24-well or 96-well plates, and cultured in the growth medium of MEM supplemented with 10% heat-inactivated FBS (MilliporeSigma), 1 mM l-glutamine, and 25 mg/L penicillin/streptomycin overnight at 37°C with 5% CO_2_ incubator. Macrophages were exposed to hydrogen peroxide (H_2_O_2_, Wako) to induce oxidatively stressed macrophages. Briefly, cells were exposed to H_2_O_2_ at 100 μM for 1 hour and at 200 μM for 1 hour in growth medium at 37°C with a 5% CO_2_ incubator with an interval of 30 minutes. Thereafter, cells were treated after exposure to 200 μM H_2_O_2_ with either 100 nM β-estradiol (MedChemExpress) or 10 μM GIC968 (Cayman Chemical), then cultured for 24 hours. Macrophages were next subjected to a Senescence β-Galactosidase staining kit for evaluating senescence β-gal activity and to protein expression analysis using Western blotting. When cells were treated with β-estradiol, FBS was substituted with charcoal-stripped FBS (SERANA).

### Immunofluorescence.

Macrophages were seeded on coverslips and then fixed by 4% paraformaldehyde solution (Wako) for 30 minutes at 4°C. Cells were permeabilized with 0.3% Triton X-100 (MilliporeSigma) in PBS for 3 minutes and then incubated with the primary antibody diluted in 3% FBS in PBS (1:100) for 1 hour at 37°C in a moist chamber. A secondary antibody of Alexa Fluor 488–conjugated goat anti-rabbit (Molecular Probes, Thermo Fisher Scientific) or anti-mouse Alexa Fluor 589–conjugated (Jackson ImmunoResearch) IgG at a dilution of 1:250 was applied to the coverslips and incubated for 30 minutes at 37°C. Hoechst was used to label the nuclear DNA of the cells (Molecular Probes, Thermo Fisher Scientific). Coverslips were mounted (Dako) and analyzed by Keyence All-in-One Microscope.

### Western blotting.

Macrophages were lysed using sample buffer EzApply (ATTO) and heated at 100°C for 5 minutes. The extracted proteins were subjected to SDS-PAGE gels and transferred electrophoretically onto PVDF membranes (Immobilon-P Membrane; Merck). The membranes were blocked in 5% skim milk and then incubated with each primary antibody at the concentration recommended in the manufacturer’s instructions. Primary antibodies included those against GAPDH (Bioss Antibodies), β-actin (Cell Signaling Technology), β-galactosidase (Invitrogen, Thermo Fisher Scientific), P53 (GeneTex), phospho-p53 (GeneTex), P21 (GeneTex), phospho-P21 (Bioss), CD52 (Invitrogen, Thermo Fisher Scientific), and IL-1β (Cell Signaling Technology). Each respective secondary antibody conjugated with HRP (BioLegend) was used for detection of bound antibodies (Supplemental Figure 17). Signals were detected by Ez WestLumi Plus (ATTO) and Quantity One v. 4.6.9 (Bio-Rad) software. Band intensities were quantified based on intensities of target/β-actin or GAPDH using ImageJ.

### Quantitative real-time polymerase chain reaction.

Macrophages were homogenized and lysed using the TRIzol Reagent. Purified RNA samples (0.5 μg) were used to synthesize cDNAs using GoScript reverse transcriptase kit (Promega) and assayed using the SYBR Premix Ex Taq II (TAKARA) with gene-specific primers (Supplemental Figure 18). Gene expression of each target was determined by the Ct^2-ΔΔ^ method.

### Statistics.

Statistical analyses were performed using the 1-way ANOVA followed by Tukey’s multiple-comparison procedure for comparing the differences among groups, and Student’s 2-tailed *t* test procedure was used for comparison the differences between 2 independent groups (GraphPad Software). Results are presented as means ± SEM and were considered statistically significant when *P* < 0.05.

### Study approval.

Procedures for animal experiments were performed in accordance with our approved protocol (22-0135) by the Institute of Animal Care and Use Committee of the Hokkaido University Graduate School of Medicine. The research protocols for human samples were approved by the Research Ethics Review Committee of Hokkaido University Hospital (Approval ID: 018-0334). Informed consent was obtained from all donors for the use in the research before total hip arthroplasty.

### Data availability.

All supporting data values are provided in the Supporting Data Values file. The RNA-Seq data included in this study are publicly available at the NCBI GEO database (https://www.ncbi.nlm.nih.gov/geo/) with an accession numbers GSE275459 for bulk RNA-Seq and GSE275976 for scRNA-Seq. The data are available within the article and the supplement, and other materials can be obtained by contacting the corresponding author.

## Author contributions

YN conceptualized the study, designed and performed experiments, analyzed and interpreted results, and wrote the manuscript. MAT conceptualized the study, designed experiments, analyzed and interpreted results, acquired and provided funding resources, supervised whole experiments, and wrote the manuscript. GM designed and performed experiments. SY, TT, YO, HI, JS, HA, T Ebata, and K Kitahara performed experiments. T Endo and DT acquired and provided funding resources. TS, MT, K Kadoya, and NI acquired and provided funding resources.

## Supplementary Material

Supplemental data

Unedited blot and gel images

Supporting data values

## Version 1 10/31/2024

In-Press Preview

## Version 2 12/06/2024

Electronic publication
