# Supplementary material for: Dynamic transcriptome analysis of osteal macrophages identifies a distinct subset with senescence features in experimental osteoporosis
Source: JCI Insight. 2024 Dec 6;9(23):e182418. doi: 10.1172/jci.insight.182418 (PMC11623942; doi:10.1172/jci.insight.182418)
Supplement: Supplemental data [file jciinsight-9-182418-s021.pdf]

Supplementary Materials for

Dynamic transcriptome analysis of osteal macrophages identifies distinct subset with senescence features in experimental osteoporosis

Yoshio Nishida *et al*.

Corresponding Author: M Alaa Terkawi. materkawi@med.hokudai.ac.jp

The PDF file include:

15 Supplementary Figures

3 Supplementary Tables

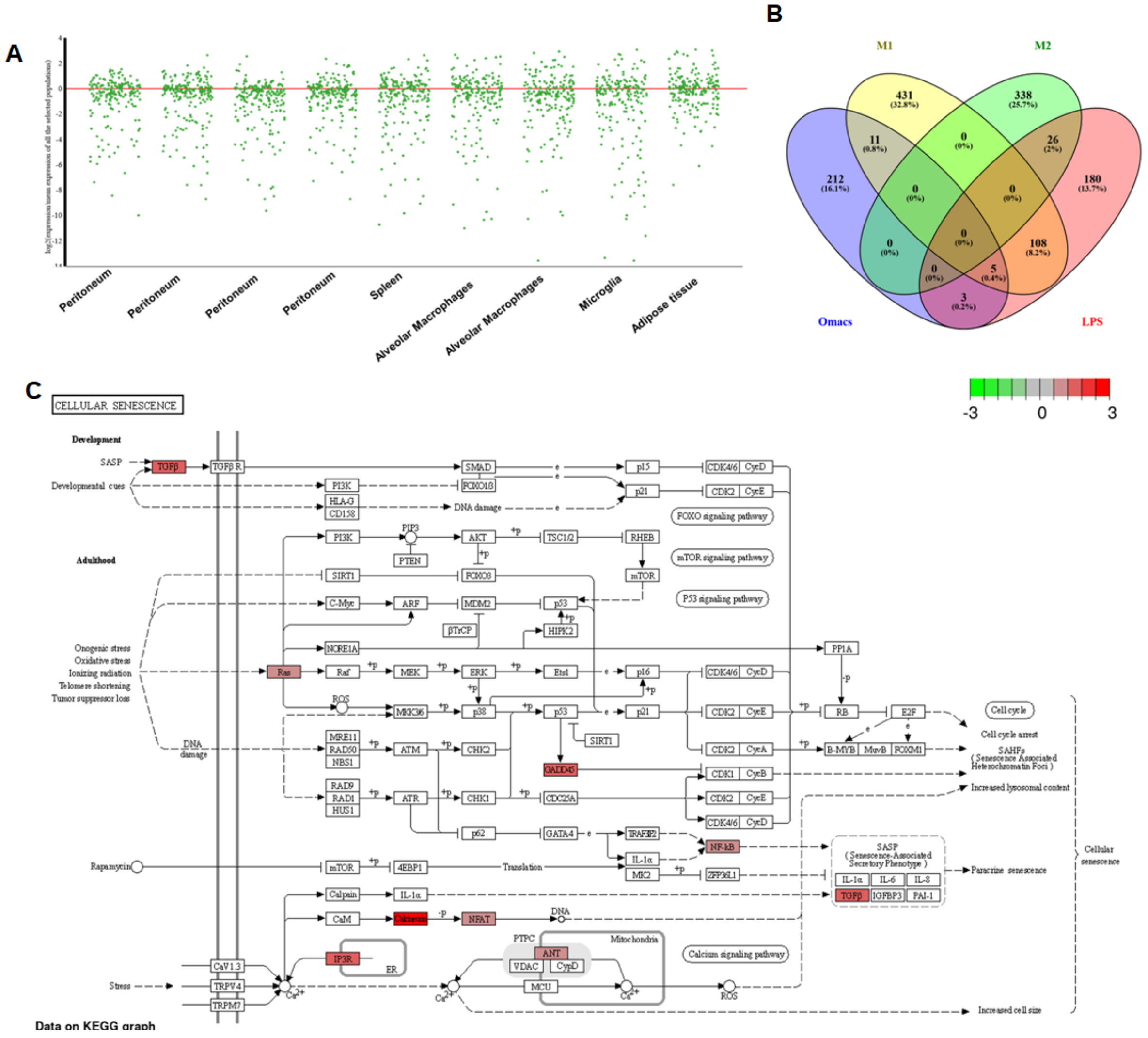

Fig. S1. Gene profiling of osteal macrophages in ovaricomized mouse assayed by bulk RNA-seq. A) W-dotplot visualizing the overlap between transcriptional signatures of OVX-Omacs and other murine macrophage populations, including lung, Kupffer cells, adipose tissue and peritoneal cavity macrophages. Red plots represent upregulated genes, blue plots represent downregulated genes, and gray plots represent not significantly regulated genes. B) Venn diagram analysis for the numbers of genes which are significantly upregulated in OVX-Omacs and an in vitro stimulated macrophage. C) Expression of the genes that are upregulated in OVX-Omacs in cellular senescence term. The analysis was made based on KEGG enrichment pathway analysis and the graph was rendered by Pathview.

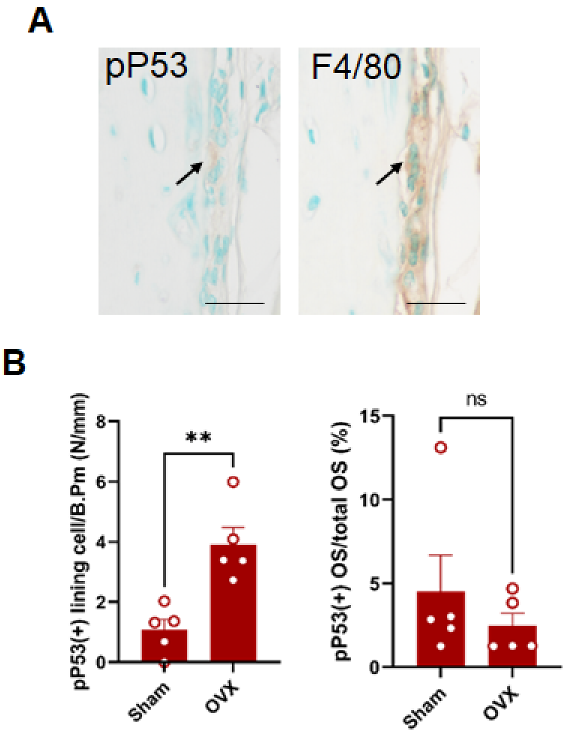

Fig. S2. Detection of pP53 in bone tissue. A) IHC examination for detection of pP53 and F4/80 in femoral bone tissue. Scale bar is 100µm. B) Percentage of pP53 stained cells in femoral bone tissue from sham and OVX mice. The positivity was counted in the lining cells and osteocytes of femoral bone tissue. Bars are the mean (n=5) ± SEM.

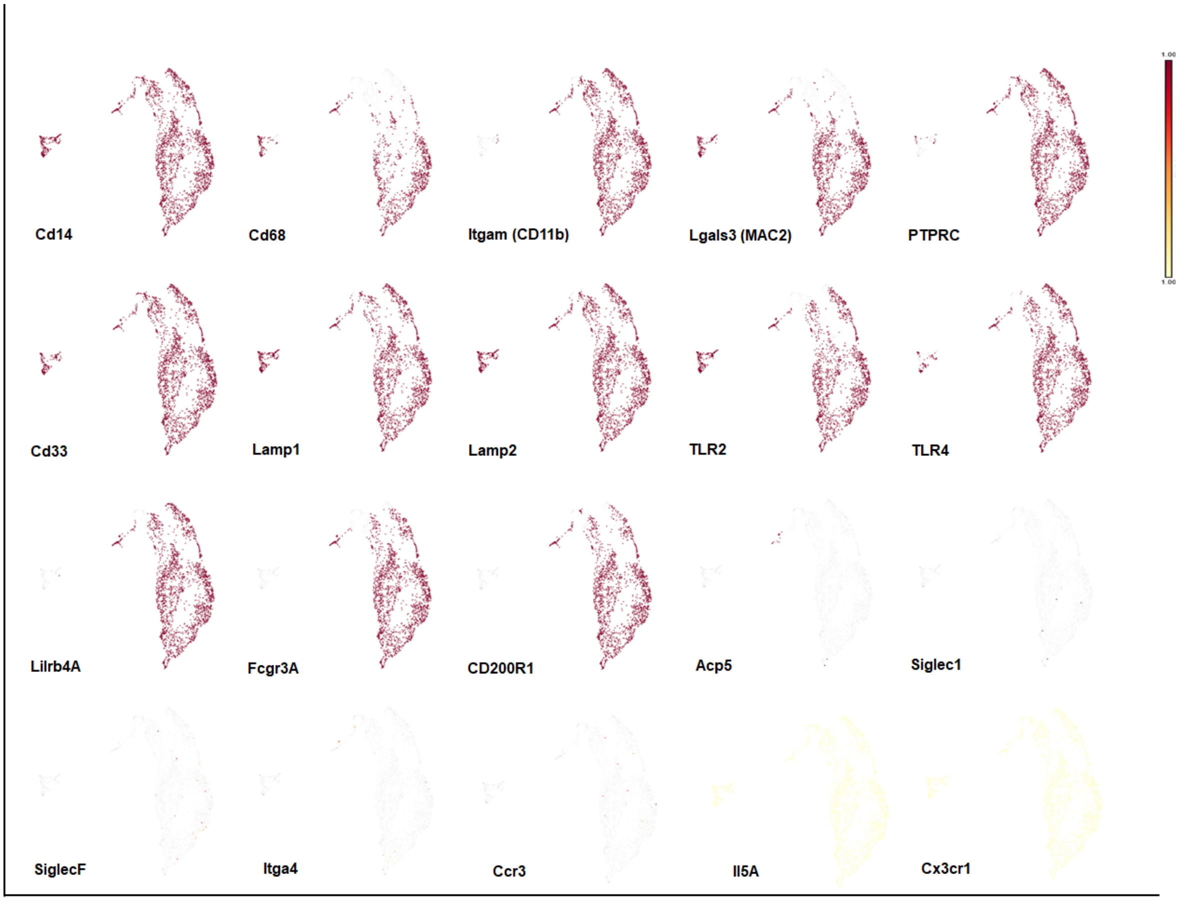

Fig. S3. UMAP plots of the scRNA-seq data. Experssion of commun tissue-resident macrophage markers not bone marrow macrophage or eosinophils in the clusters of Omacs.

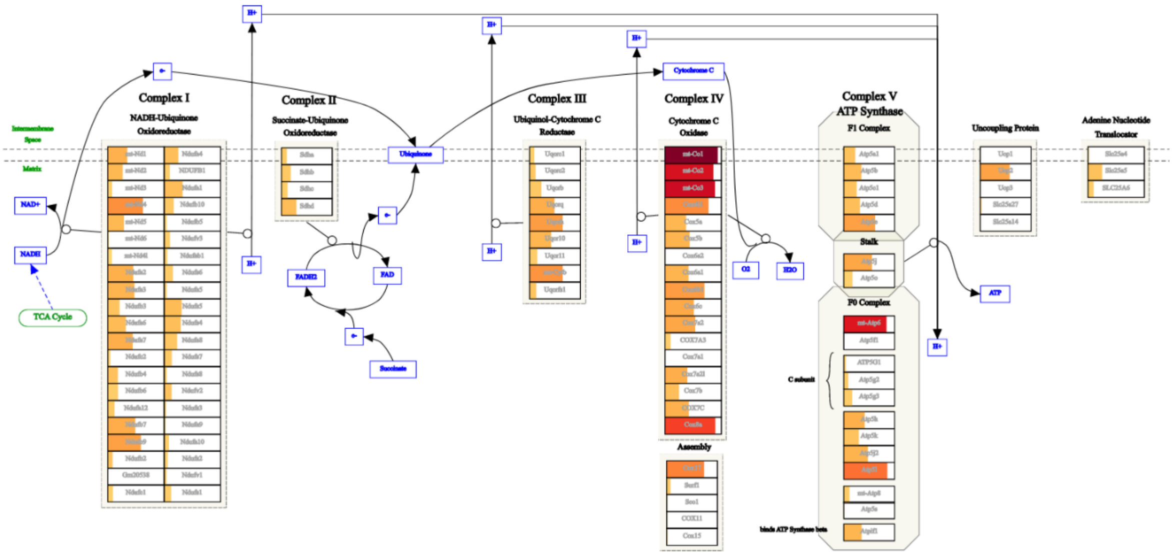

Fig. S4. Expression of the differentially expressed genes in OVX-Omacs in electron transport chain in oxidative stress term. The graph was rendered by BioTuring sofftware.

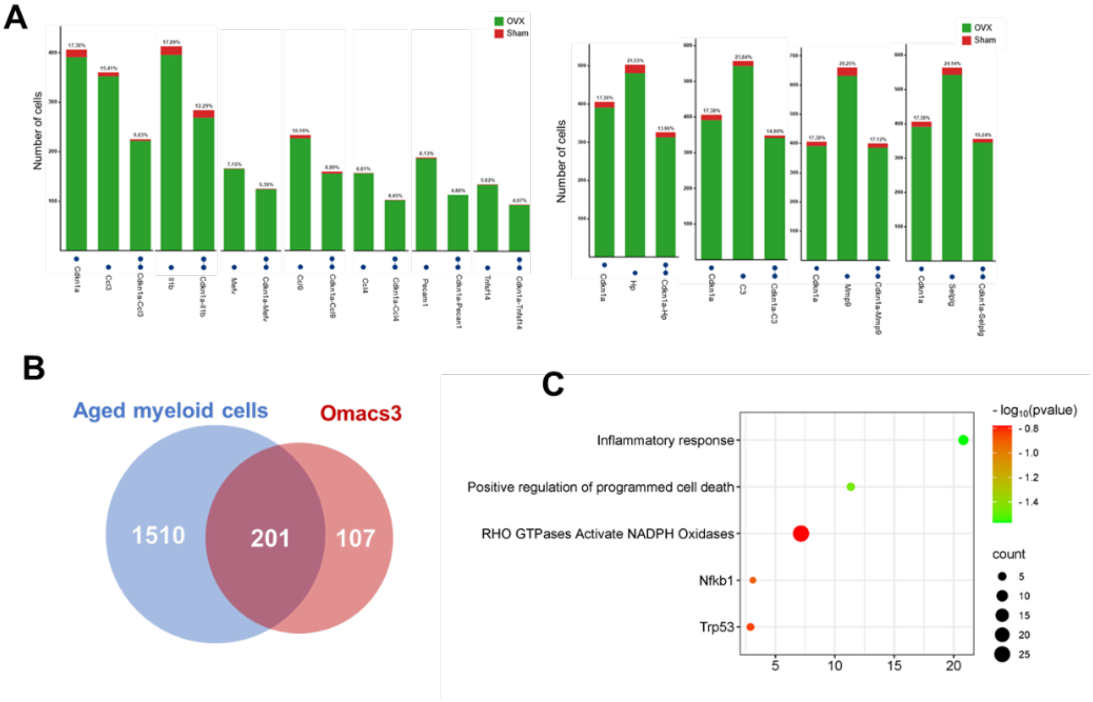

Fig. S5. Molcular characterizations of the Omacs3 from OVX mice. A) Number of cells expression of senescence markers in Omacs3 in sham and OVX mice. Markers were selected based on SenNET database for senescence markers. B) Venny analysis for differentially expressed genes in Omacs3 and these expressed in aged myeloid cells (GSE145562) from 4 organs. C) Gene enrichment analysis of genes that are differentially expressed genes in Omacs3 and aged myeloid cells (201 genes).

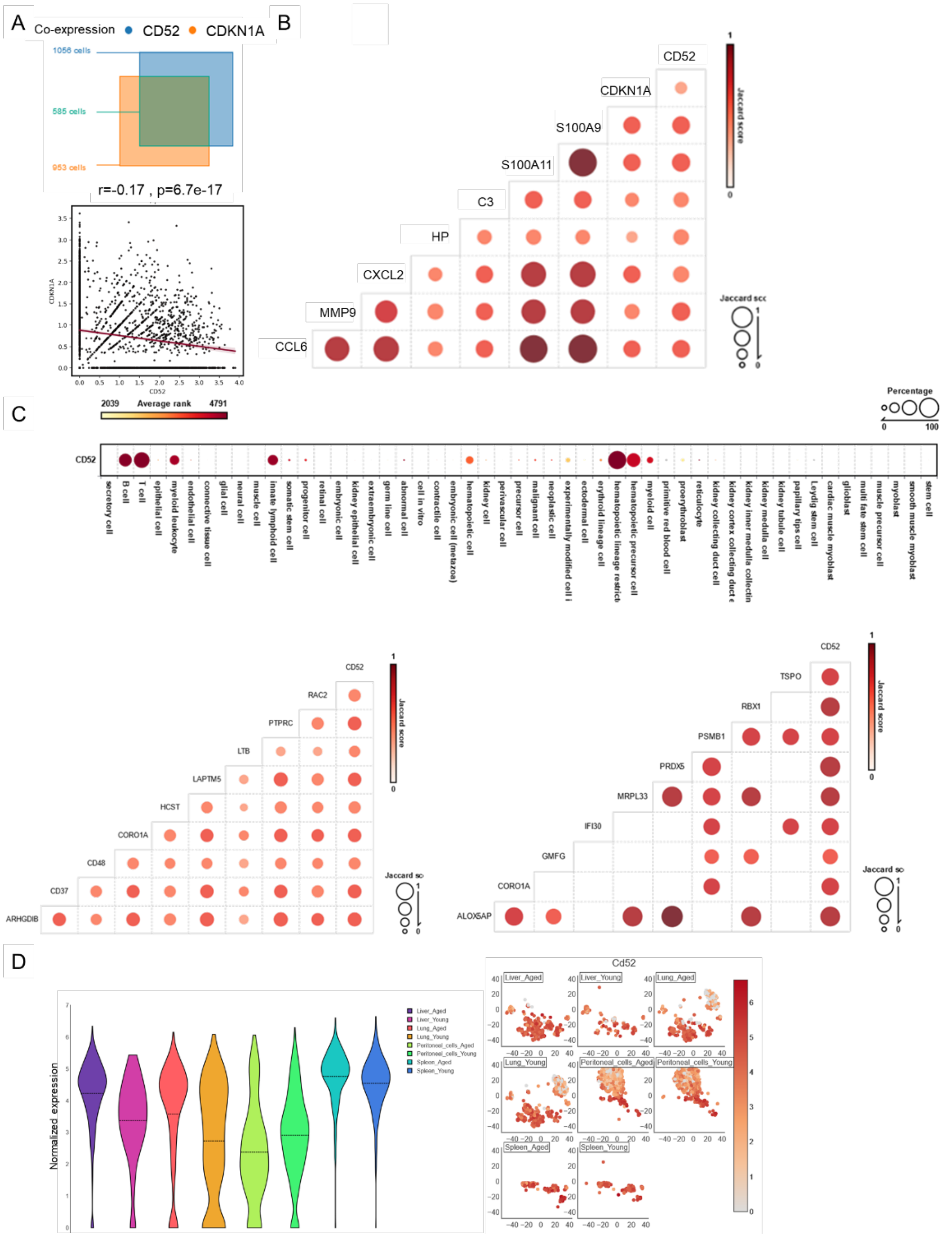

Fig. S6. Molcular characterizations of cells expressing CD52. A) Co-expression of Cd52 with Cdkn1A and analyzed by correlation coefficient test. Jaccard coefficient analysis for the genes set co-expressed in Cdkn1A expressing cells. B) Co-expression analysis of Cd52 and senescence markers in Omacs. C). Expression of Cd52 in different murine cells (upper panel) and co-expression analysis (lower panel) from public databases, as right panel for all Cd52 expressing cells and left panel for only myeloid cells. D) Expression of Cd52 in aged myeloid cells from 4 organs shown as violin plot (left panel) and UMAP plot of color-coded cell cluster (right panel).

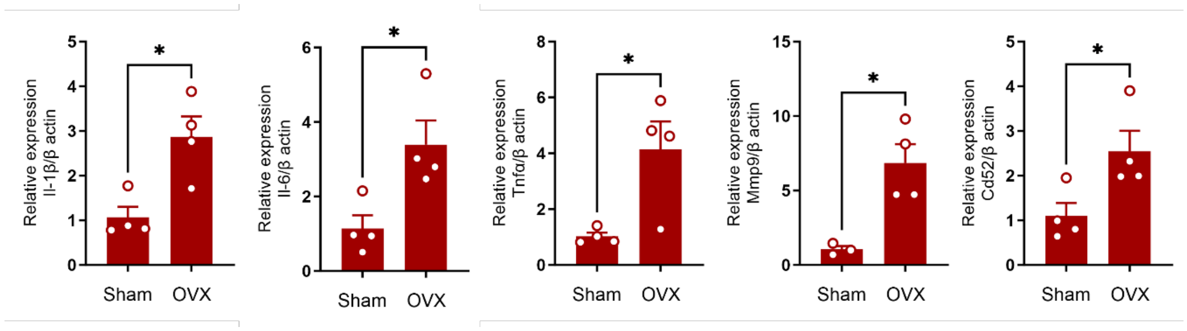

Fig. S7. Gene expression analysis of senescence markers in peritoneal macrophages of sham and OVX mice by qRT-PCR. Results represent relative expression values of each target gene to the expression of β-actin. Results represent means of 4 samples ± SEM and significant difference was determined by the two-tailed Student's *t* test.

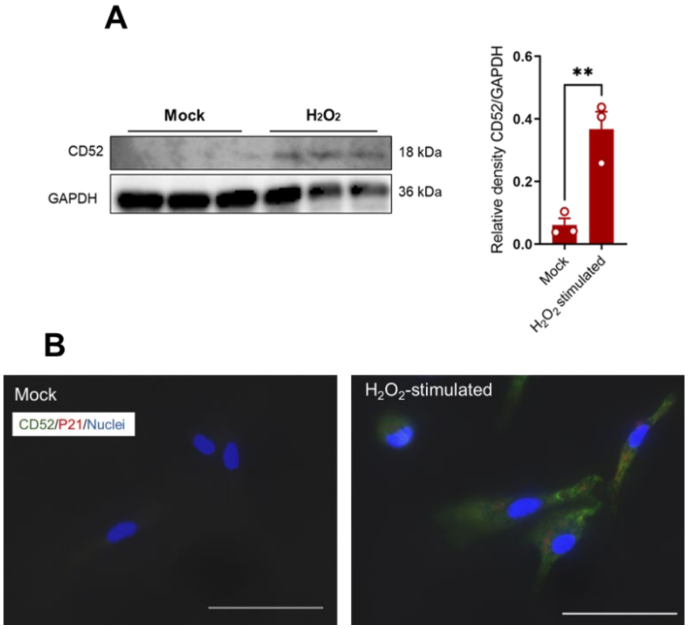

Fig. S8. Expression of CD52 in peritional macrophages exposed to H_2O_2-induced oxidative stress. A) Western blotting results of stimulated macrophages. The left panel represents blots analysis with antibodies and right panel represents quantification of band density for each target. Results represent means of 3 samples ± SEM and significant difference was determined by the two-tailed Student's *t* test. B) Immunofluorescence test of stimulated macrophages as stained by antibodies to CD52 and P21. Scale bars are 50µm.

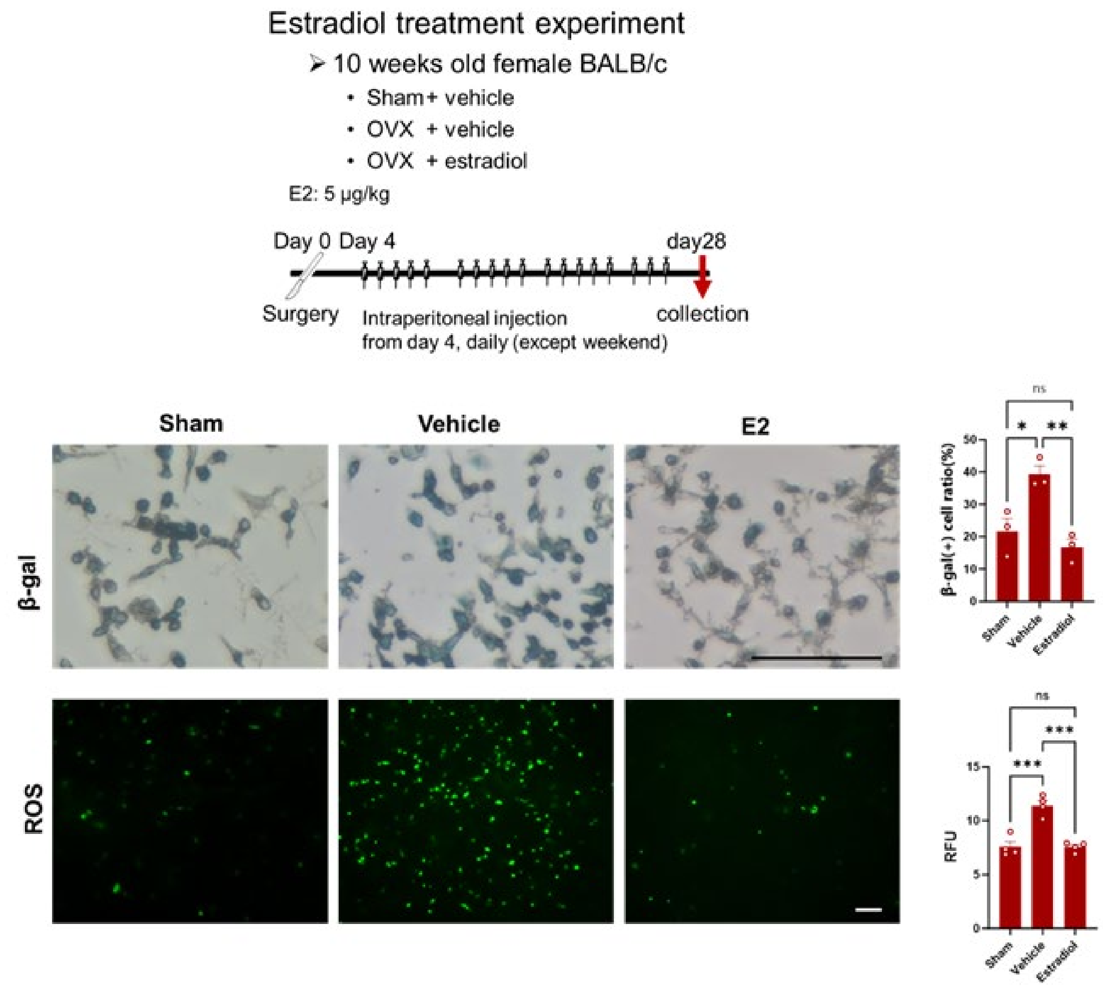

Fig. S9. Effects of estradiol treatment on development of senescent macrophages. Upper panel shows the threatmnet procedure. Lower panels resprtesnt number of positive peritional macrophages to β-gal and ROS in OVX mice. The left panels are representative images of stained cells. The right panels show quantification analysis. Scale bars are 100μm. Results represent means of 3 and 4 samples ± SEM and significant difference was determined by the one-way ANOVA, followed by Tukey's multiple-comparison procedure. * = $P < 0.05$; ** = $P < 0.01$; *** = $P < 0.001$; ns: not significantly different.

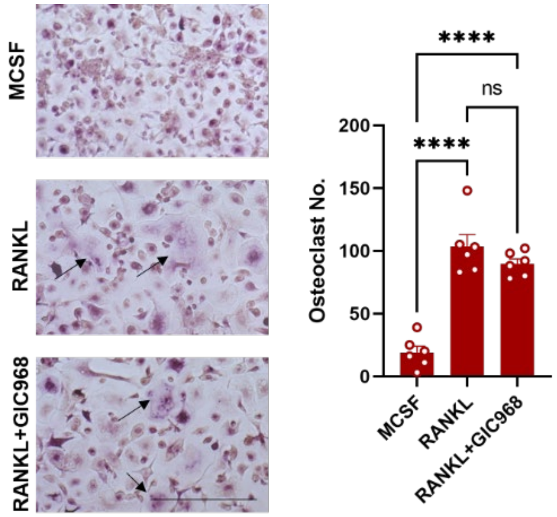

Fig. S10. Effects of GIC968 on the number of differentiated osteoclasts in vitro. Left panels show representative images for each treatment. Raight panels show the quantification of TRAP+ cells. Scarle bar is 100µm. Results represent the count of positive cell ± SEM (n=6). The significant difference was determined by the one-way ANOVA, followed by Tukey's multiple-comparison procedure.

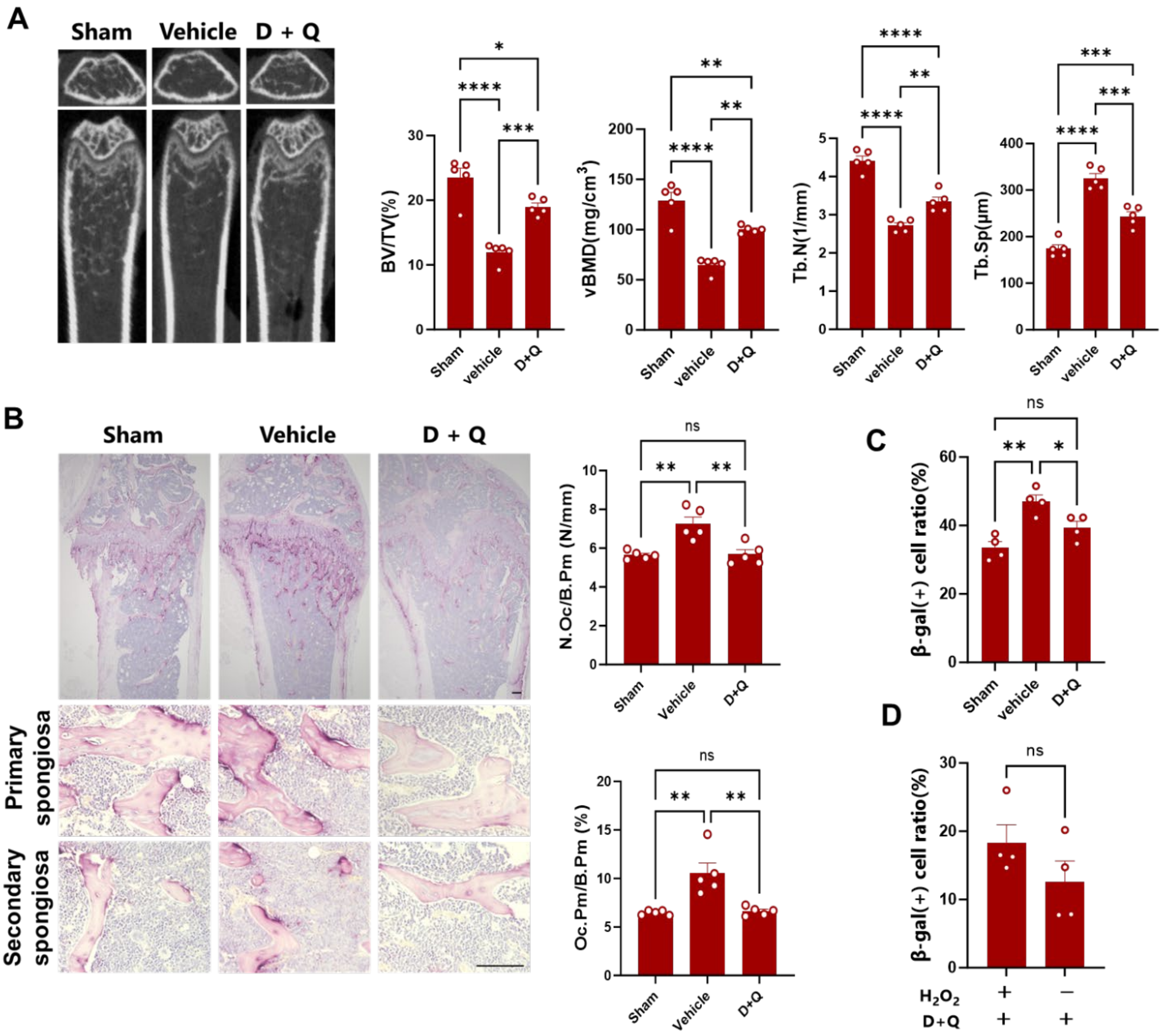

Figure S11. Therapeutic effects of dasatinib and quercetin in experimental osteoporosis. A) Representative micro-CT images of femoral bones in OVX mice. The right panels show bone parameters including BV/TV, vBMD, Tb.N, and Tb.Sp of femoral bones. B) TRAP-stained sections of femoral bone, including primary and secondary spongiosa. Scale bars are 100 μm. The right panels show quantification of osteoclasts number and sizes on the surface of femoral bone. Results represent means of 5 samples ± SEM and significant difference was determined by the one-way ANOVA, followed by Tukey's multiple-comparison procedure. * = $P < 0.05$; ** = $P < 0.01$; *** = $P < 0.001$; **** = $P < 0.0001$; ns: not significantly different. C) Percentage of β-gal-positive macrophages collected from peritoneal cavity. D) Percentage of β-gal-positive macrophages stimulated by H_2O_2 with or without D and Q. Results represent means of 4 samples ± SEM and significant difference was determined by the two-tailed Student's *t* test.

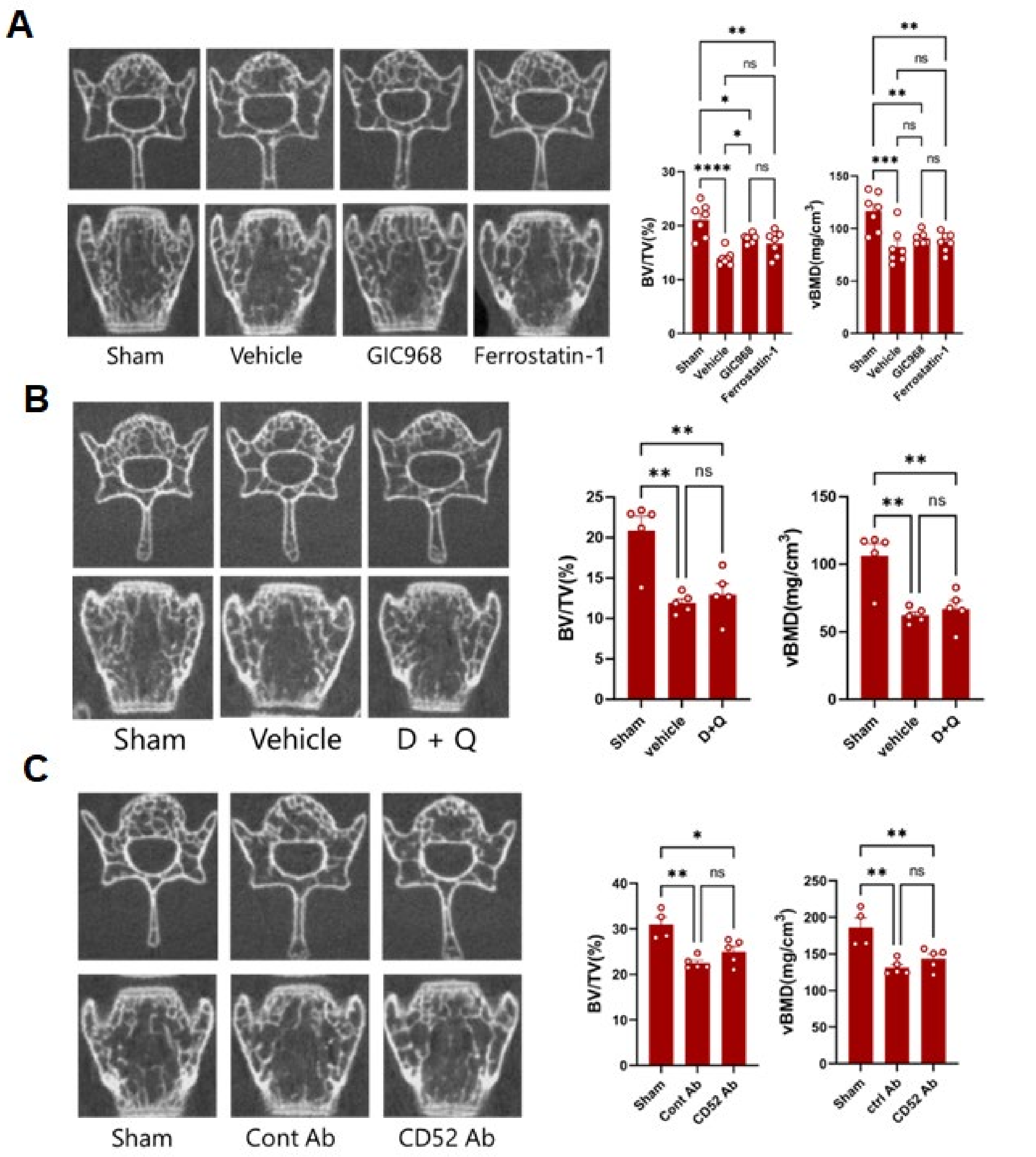

Fig. S12. Beneficial effects of elimination of senescent macrophages in postmenopausal osteoporosis model. A) For treatment with GIC968 and Ferrostatin-1. B) For treatment with dasatinib and quercetin. C) For treatment with CD52 antibody. Left panels are representative micro-CT images of vertebral bones of the mice. Right panels show major bone parameters including BV/TV, and vBMD. Bars are the mean 7 and 5± SEM. The significant difference was determined by the one-way ANOVA, followed by Tukey's multiple-comparison procedure.

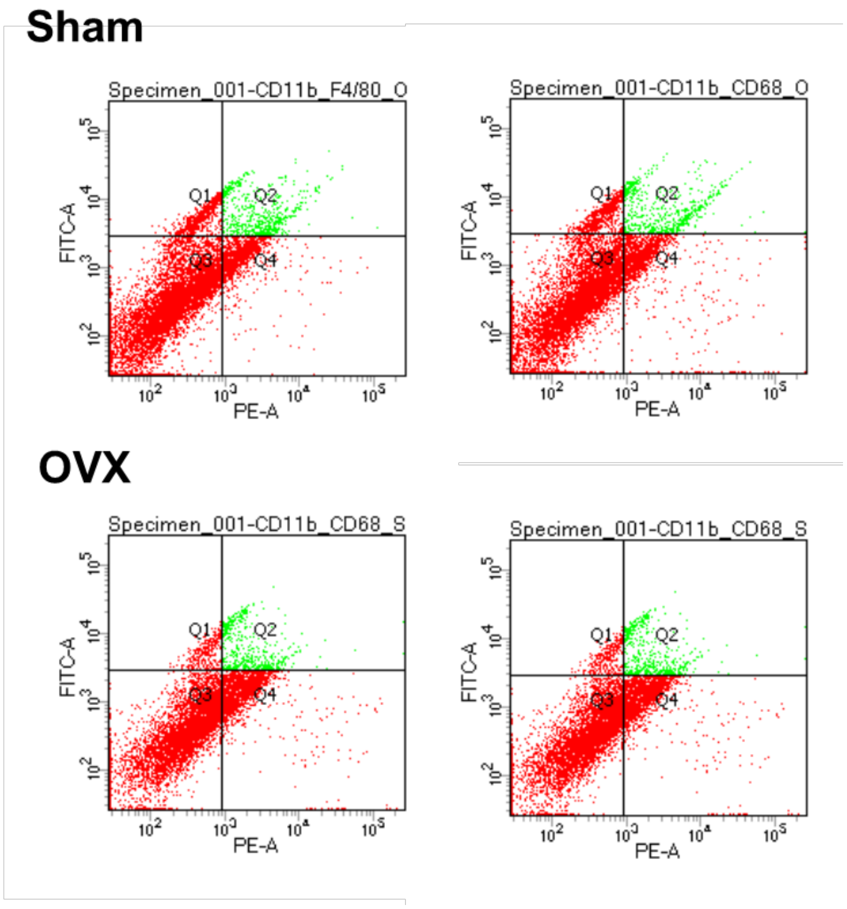

Fig. S13. Gating strategy for separation of osteal macrophages using fluorescence-activated cell sorting targeting CD11b+CD68 or F4/80 positive cells. Cells were isolated and further subjected to RNA-seq analyses.

Table S14. Reagents used for performing scRNA-seq data and analysis.

**Hashtag / CITE-seq**	Hashtag (Yes/No)	Yes
	CITE-seq (Yes/No)	No
	Antibodies Used:	TotalseqB 0301, 0302
**Single-cell Library Preparation**	GEM Kit/PN#	Next GEM Single Cell 3′ GEM Kit v3.1/PN-1000123
	Library Kit/PN #	Next GEM Single Cell 3′ Library Kit v3.1/PN-1000158
	Gel Beads Kit/PN #	Next GEM Single Cell 3′ Gel Bead Kit v3.1/PN-1000129
	Next GEM Chip/PN #	Next GEM Chip G Single Cell Kit/PN-1000120
	Index Kit/PN #	Single Index Kit T Set A/PN-1000213
	Thermal Cycler Instrument	ThermoFisher ProFlex PCR Sytem
	cDNA QC Instrument	Perkin Elmer LabChip
	Library QC Instrument	Perkin Elmer LabChip
	PCR Cycles for cDNA	12
	cDNA Concentration (ng/uL)	1.35
	PCR Cycles for Library	14
	library concentration (ng/uL)	18.3
**Sequencing**	Sequencer	MGI DNBSEQ-G400
	Flowcell Type	DNBSEQ-G400RS High-throughput Sequencing Set (App-A FCL PE100)
	R1 Sequencing Cycles (cycle)	28
	i7 Index (cycle)	8
	i5 Index (cycle)	-
	R2 Sequencing Cycles (cycle)	100
	Number of reads	435,107,683
**Cell Ranger**	Cellranger Version	cellranger-6.0.0
	Reference Genome	mm10-2020-A
	Total Number of Cells Recovered	2349
	Mean Reads/Cell	185231
	Median Genes/Cell	1203
	Median UMIs/Cell	4387

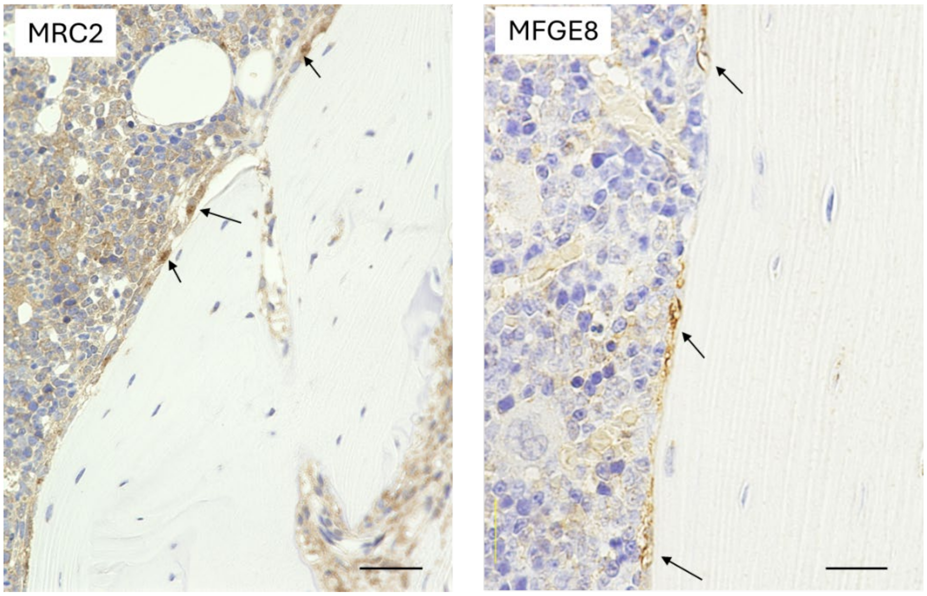

Fig. S15. Detection of Omacs 1 and 2 in bone lining cells using specific antibodies. Bone tissues were stained by MRC2 and MFGE8 antibodies. Arrows indicate positive-stained cells. Scale bars are 50 μm.

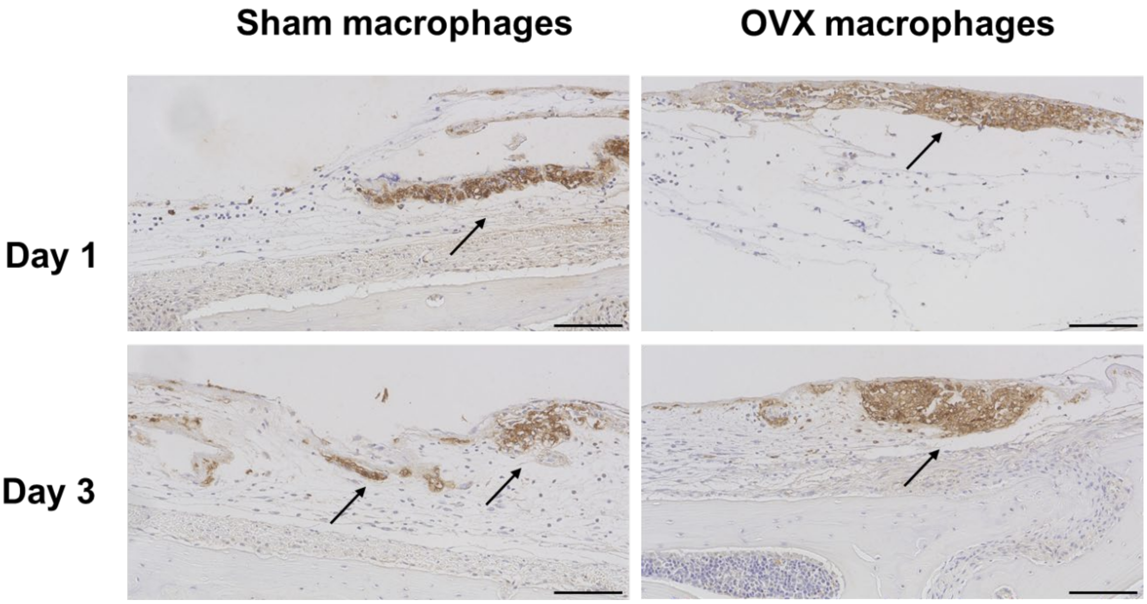

Fig. S16. Detection of macrophages grafted on calvarial bone. GFP-positive cells were stained by immunohistochemistry using specific antibody. Grafted cells were from sham and OVX of GFP-transgenic miceSampling was performed on day 1 and day 3 post-grafting. Arrows indicte the positive-stained cells. Scale bars are 100 μm.

Table S17. Antibodies list used in this study.

Antibody	Dilution	Manufacturer	Research Resource Identifier (RRID)
**CD11b FITC**	1:100	BioLegend	AB_312788
**F4/80 PE**	1:100	BioLegend	AB_2832546
**CD68 PE**	1:100	BioLegend	AB_10613469
**CD169 FITC**	1:100	BioLegend	AB_2563106
**MFGE8**	1:100	Invitrogen	AB_2855366
**MRC2**	1:100	EnoGene Biotech	—
**GFP**	1:1000	Invitrogen	AB_221570
**P21**	1:1000	GeneTex	AB_ 2888121
**pP21**	1:1000	Bioss	AB_ 11097560
**P53**	1:1000	GeneTex	AB_1952339
**pP53**	1:1000	GeneTex	AB_2886801
**IL-1β**	1:1000	Cell signaling	AB_2715503
**β-Galactosidase**	1:1000	Invitrogen	AB_ 2851905
**CD52**	1:1000	Invitrogen	AB_2901327
**GAPDH**	1:1000	Bioss	AB_10856675
**β actin**	1:1000	Cell Signaling	AB_330288
**Anti-rabbit HRP**	1:3000	BioLegend	AB_2099368
**Anti-mouse HRP**	1:3000	BioLegend	AB_315009

Table S18. Primer list used in this study.

Target	Forward	Reverse
β-actin	GGCTGTATTCCCCTCCATCG	CCTTCTGACCCATTCCCACC
Il-6	TAGTCCTTCCTACCCCAATTTCC	TTGGTCCTTAGCCACTCCTTC
Il-1β	TGAAATGCCACCTTTTGACAGTG	AGCCCTTCATCTTTTGGGGTC
Tnf-α	AGCCGATGGGTTGTACCTT	ATAGCAAATCGGCTGACG
Mmp-9	GCCAGCCGACTTTTGTGGTC	GTGTCCGTGAGGTTGGAGGT
Cd52	CTTGGGACAAGCCACTACGG	TGGATGAGGCCCCACTCTTT
