# Supplementary material for: Dynamic transcriptome analysis of osteal macrophages identifies a distinct subset with senescence features in experimental osteoporosis
Source: JCI Insight. 2024 Dec 6;9(23):e182418. doi: 10.1172/jci.insight.182418 (PMC11623942; doi:10.1172/jci.insight.182418)
Supplement: Unedited blot and gel images [file jciinsight-9-182418-s022.pdf]

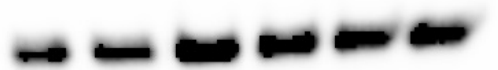

Full unedited gel for Figure 4E β-actin

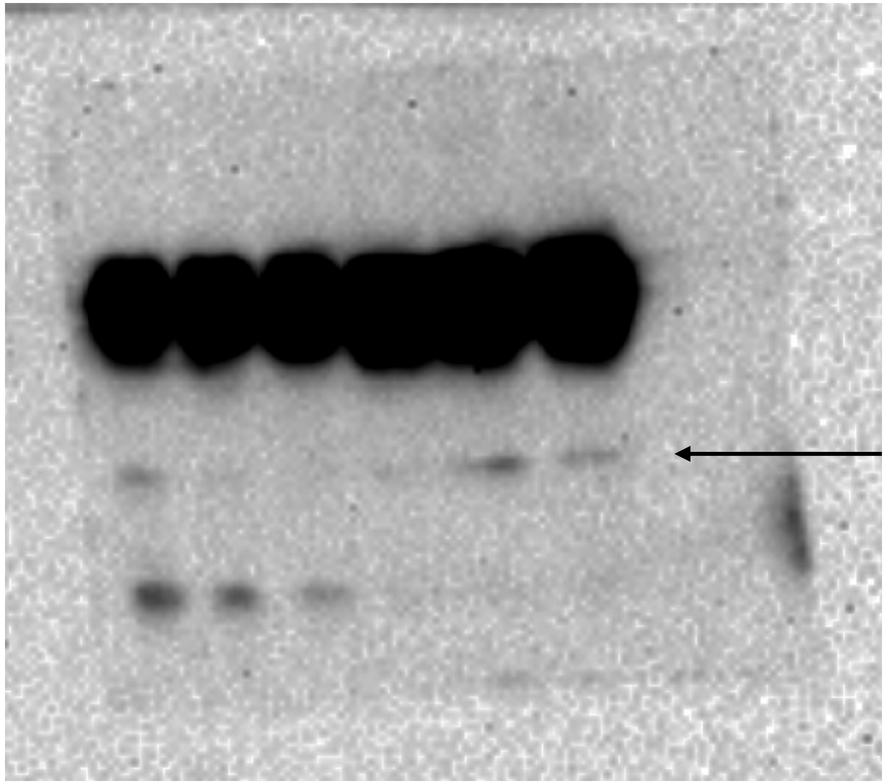

Full unedited gel for Figure 4E IL-1β

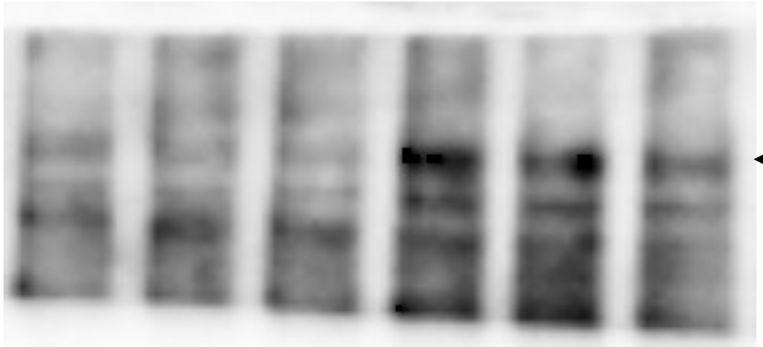

Full unedited gel for Figure 4E β-gal

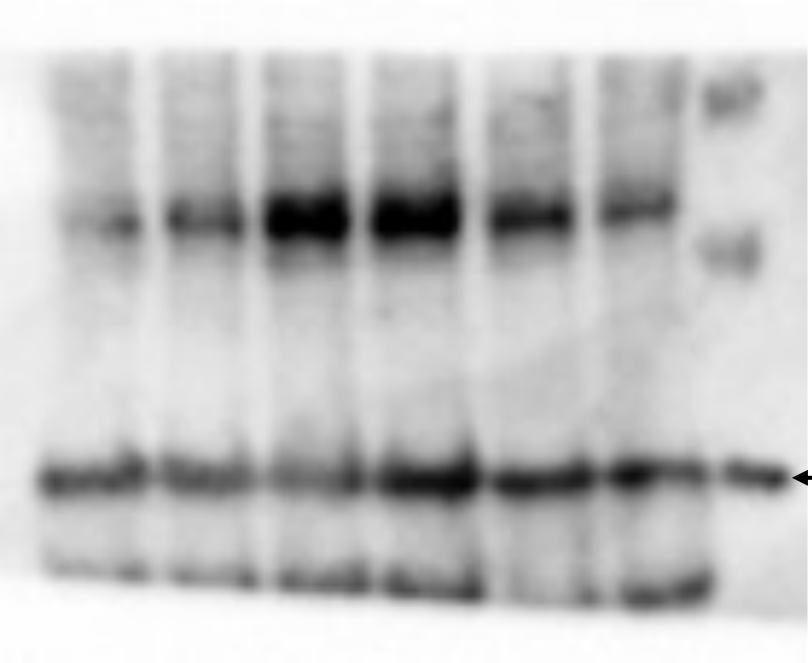

Full unedited gel for Figure 4E P21

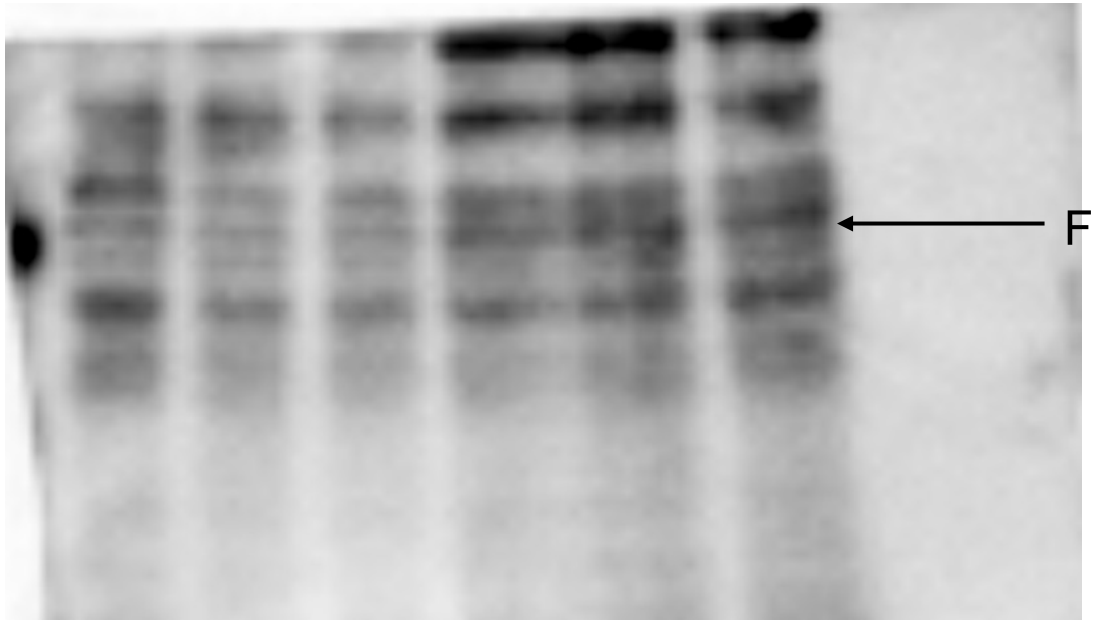

Full unedited gel for Figure 4E pP21

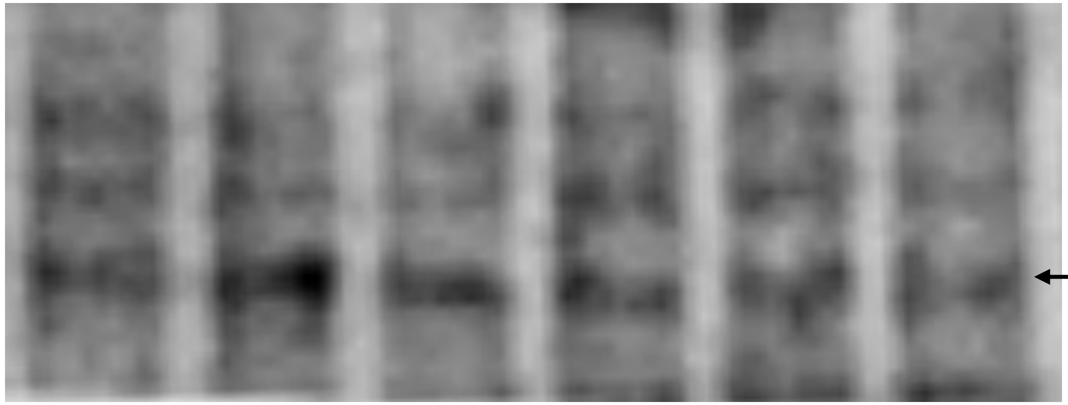

Full unedited gel for Figure 4E P53

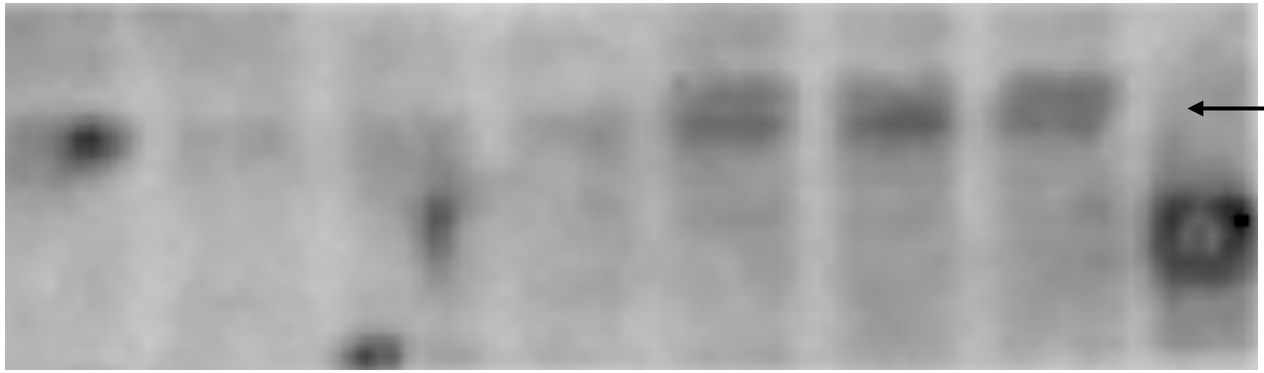

Full unedited gel for Figure 4E pP53

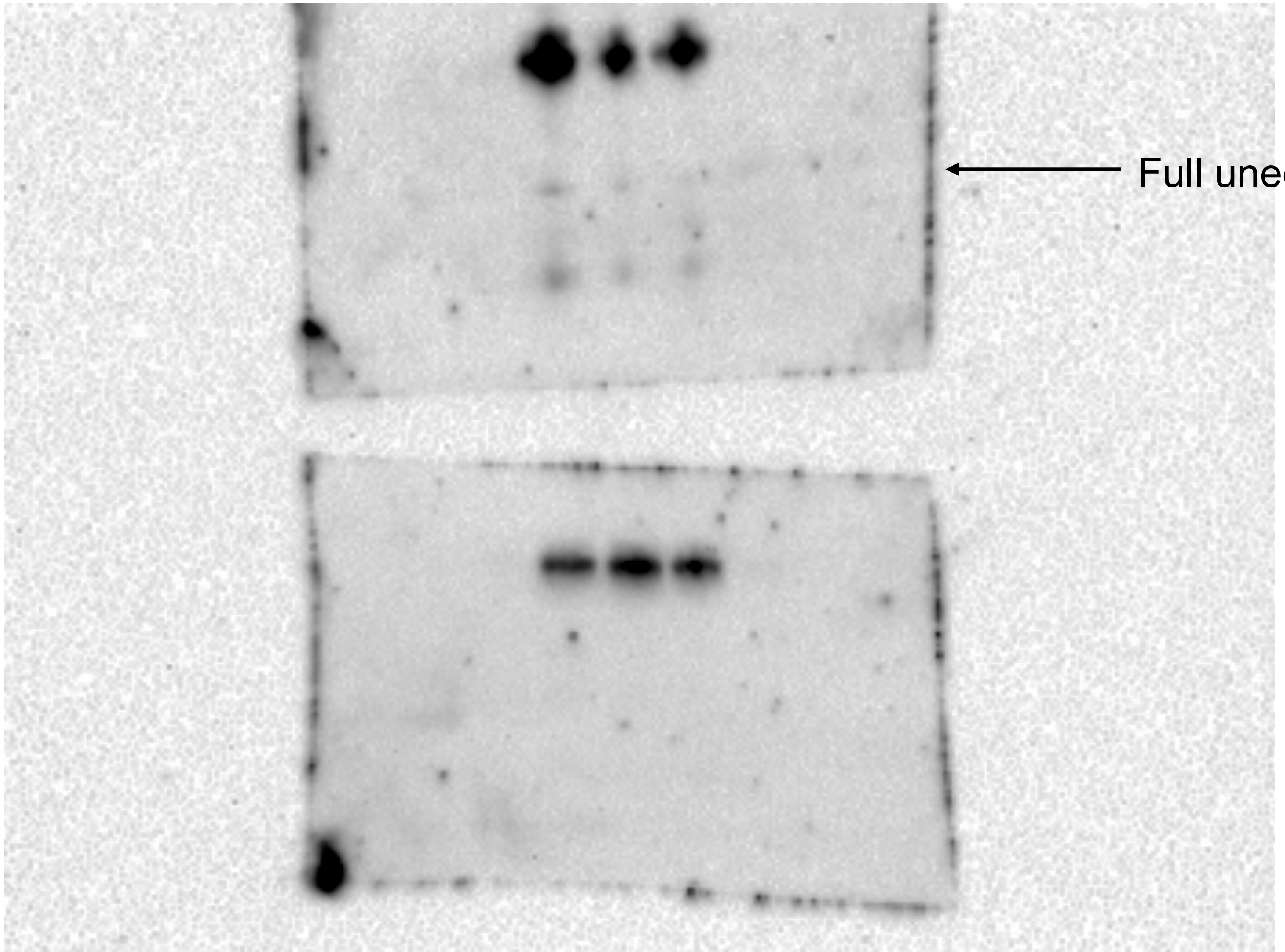

Full unedited gel for Figure 4H IL-1β

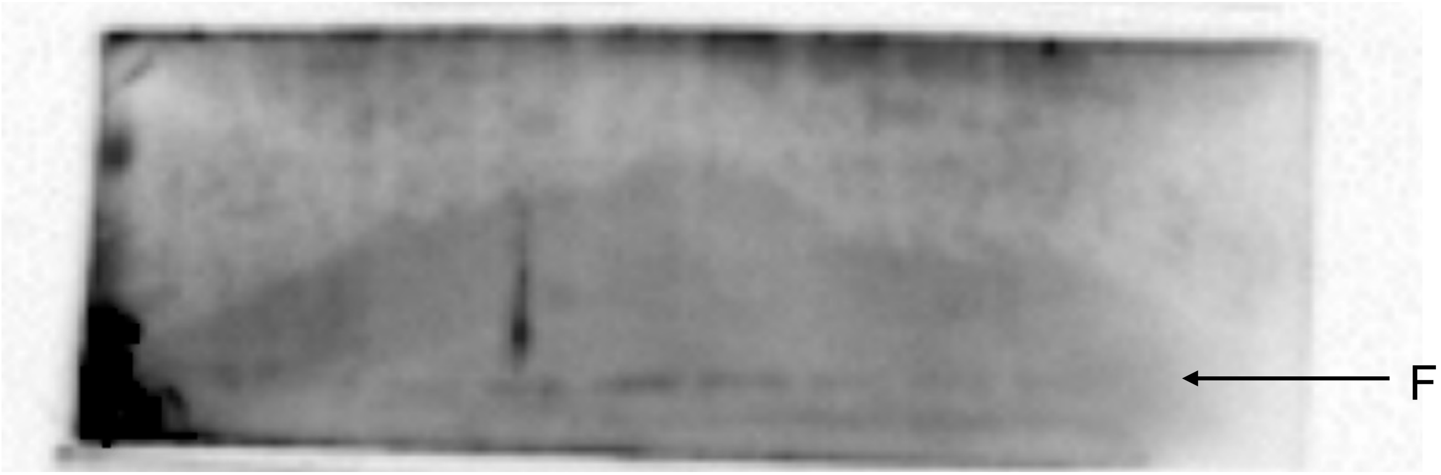

Full unedited gel for Figure 4H pP21

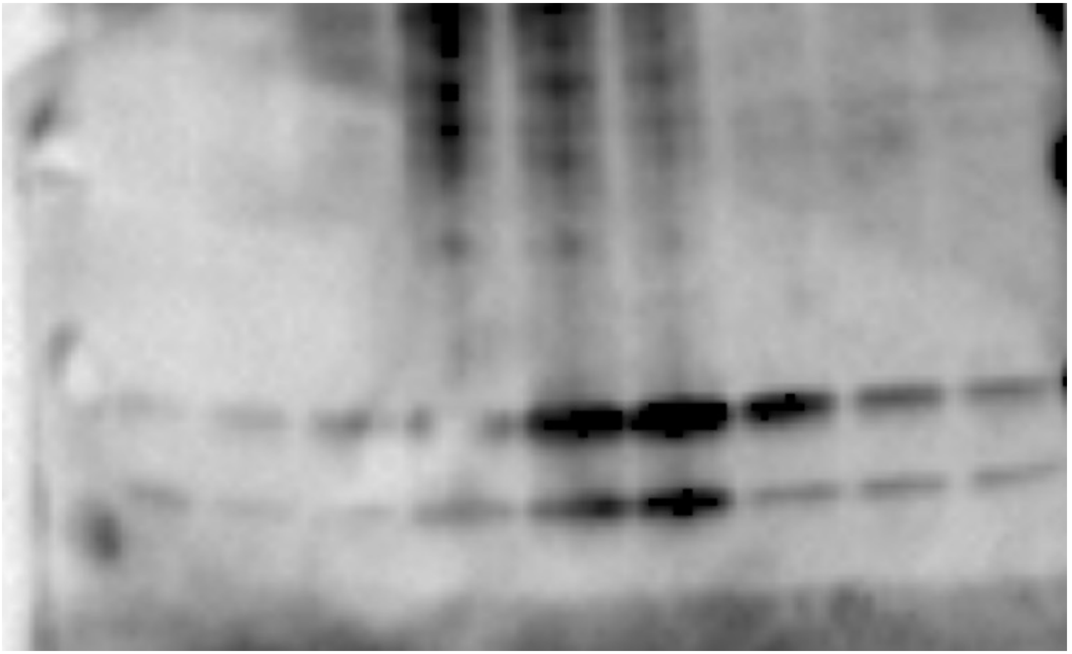

Full unedited gel for Figure 4H pP53

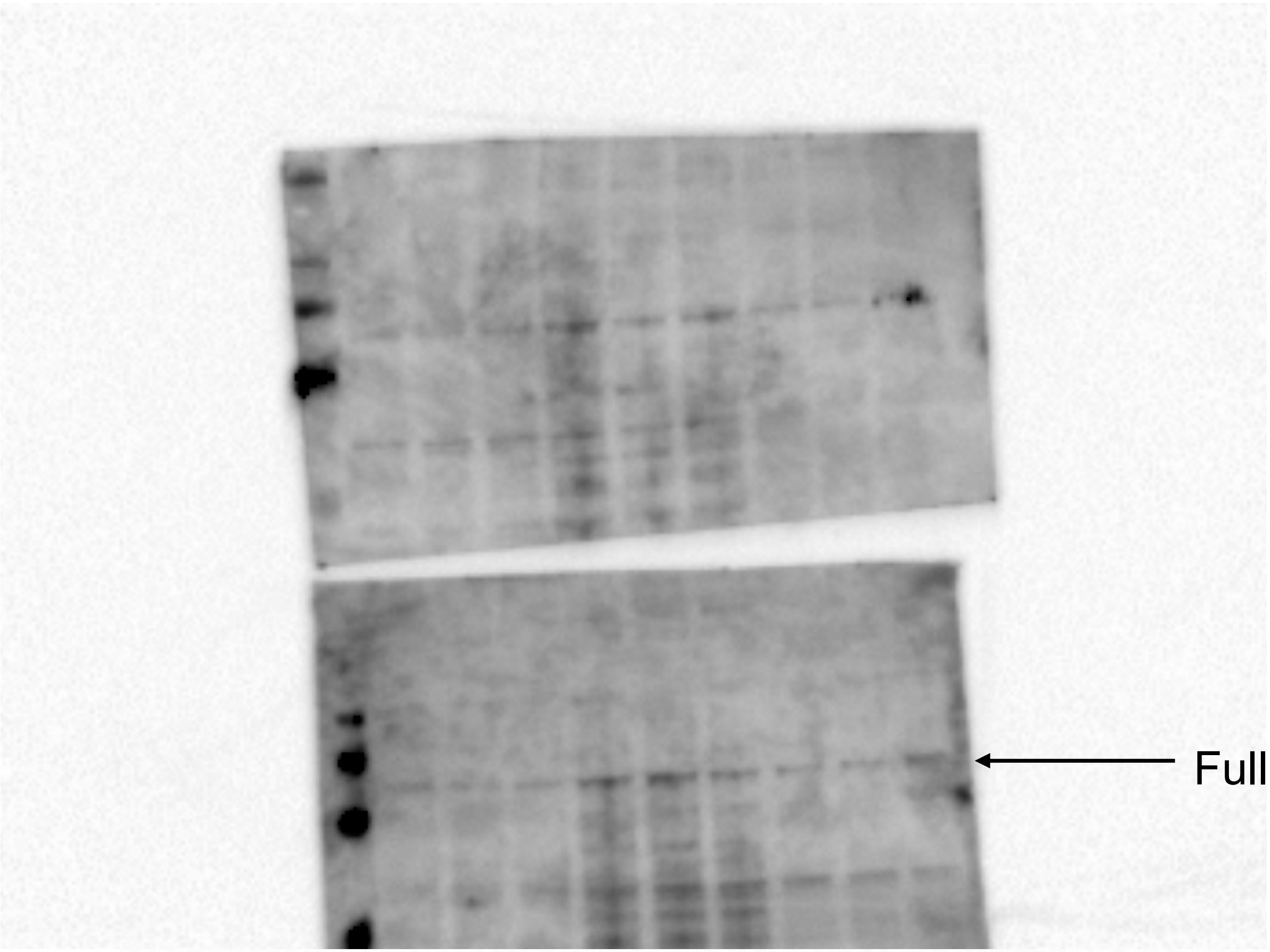

Full unedited gel for Figure 4H β-gal

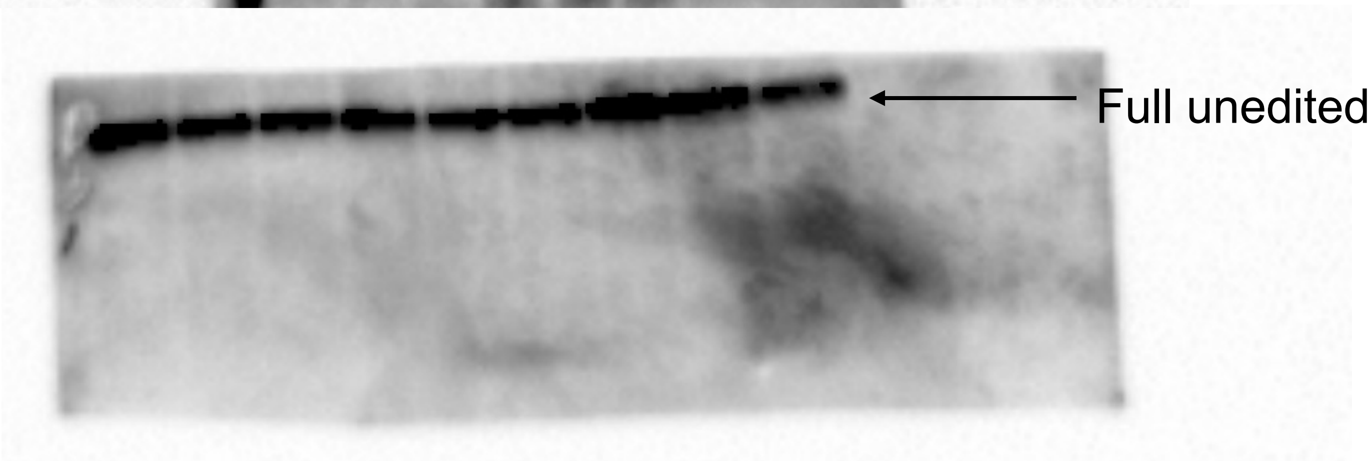

Full unedited gel for Figure 4H GAPDH

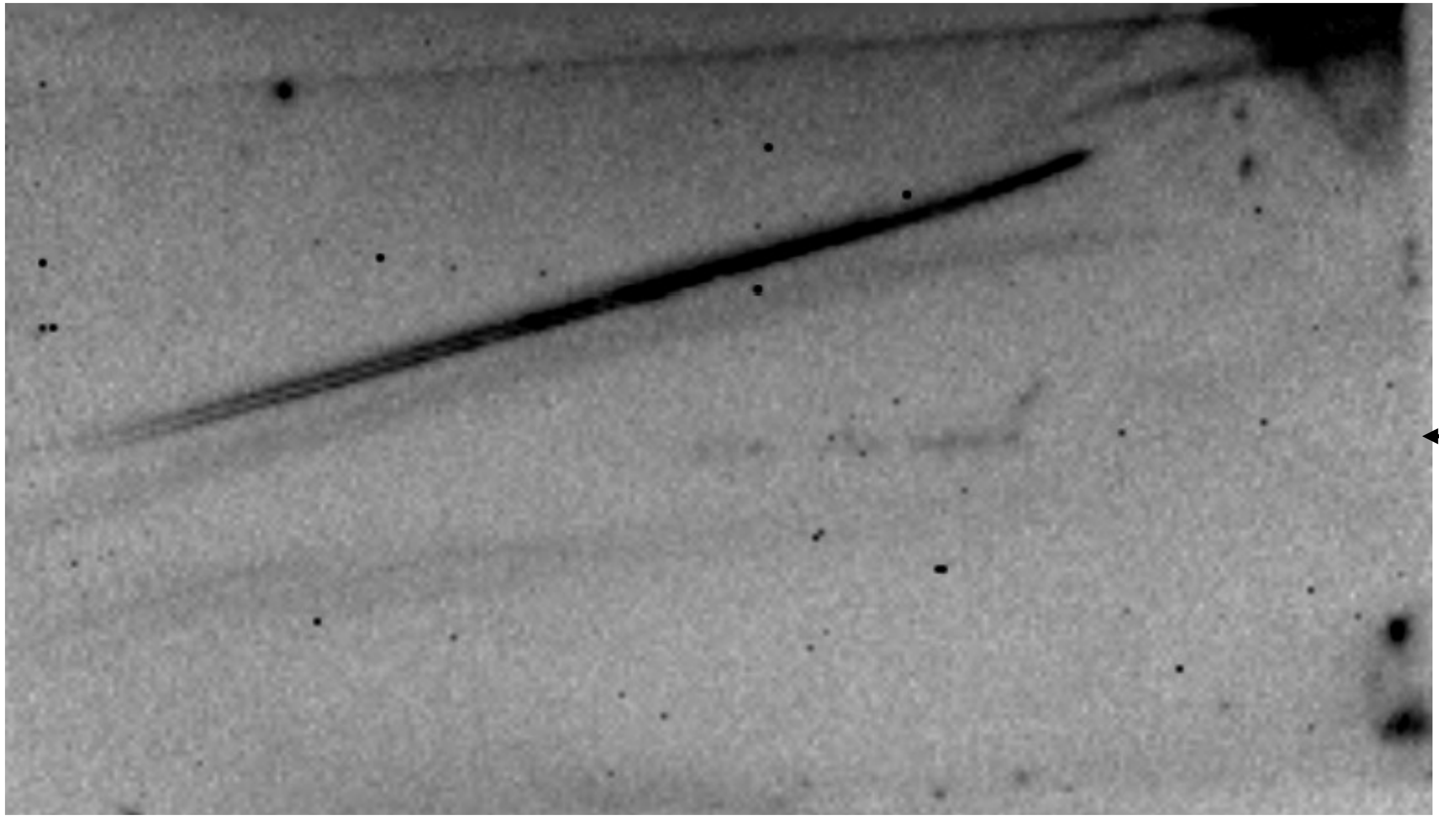

Full unedited gel for Figure 6G IL-1β

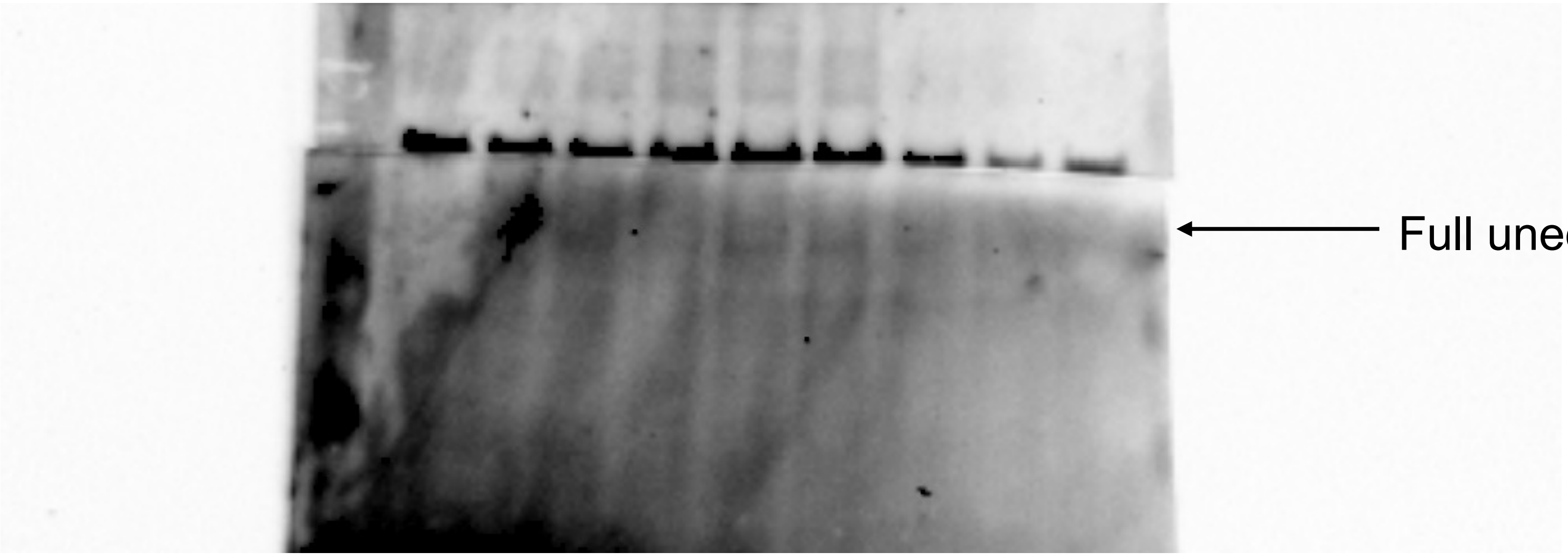

Full unedited gel for Figure 6G pP21

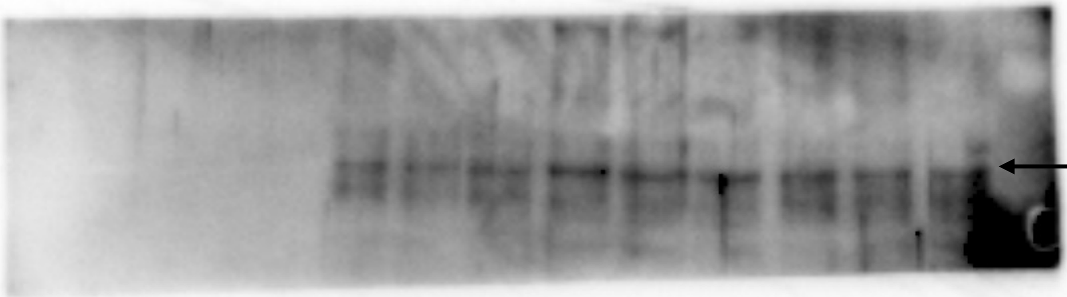

Full unedited gel for Figure 6G pP53

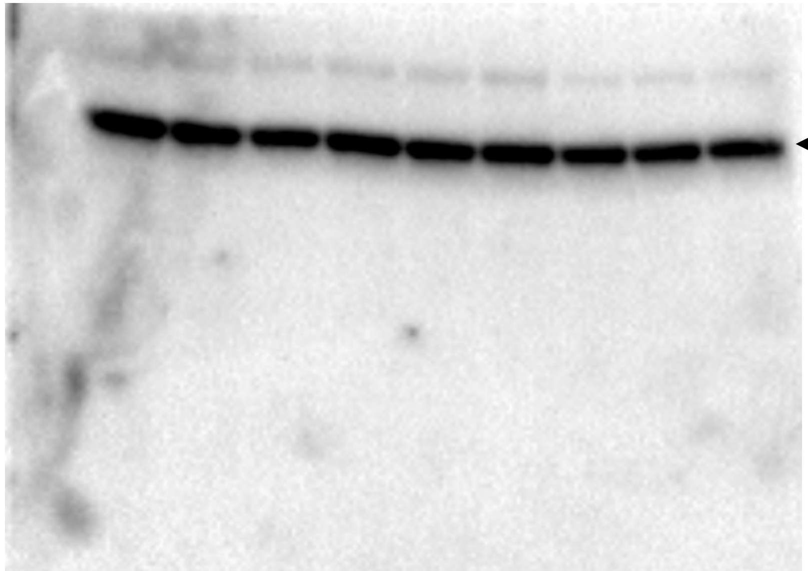

Full unedited gel for Figure 6G GAPDH

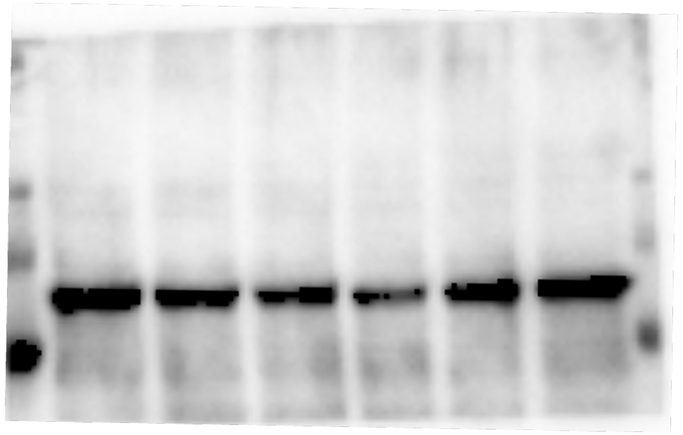

Full unedited gel for Figure 7D GAPDH

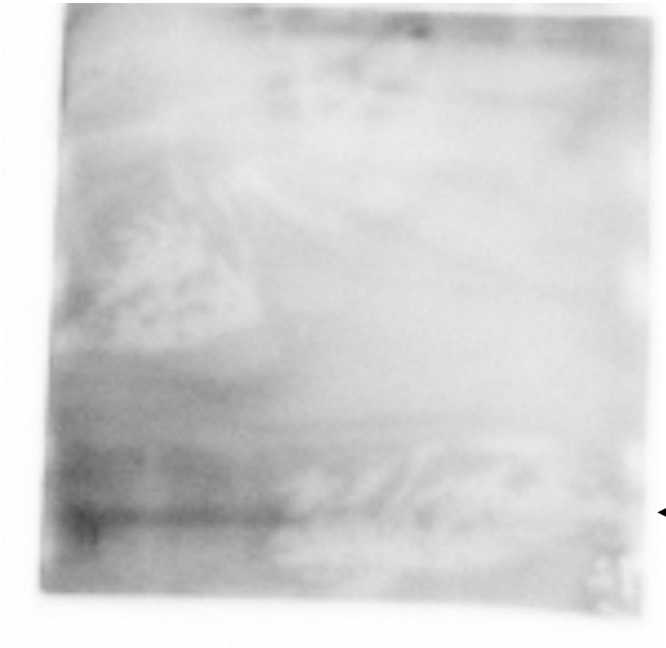

Full unedited gel for Figure 7D CD52

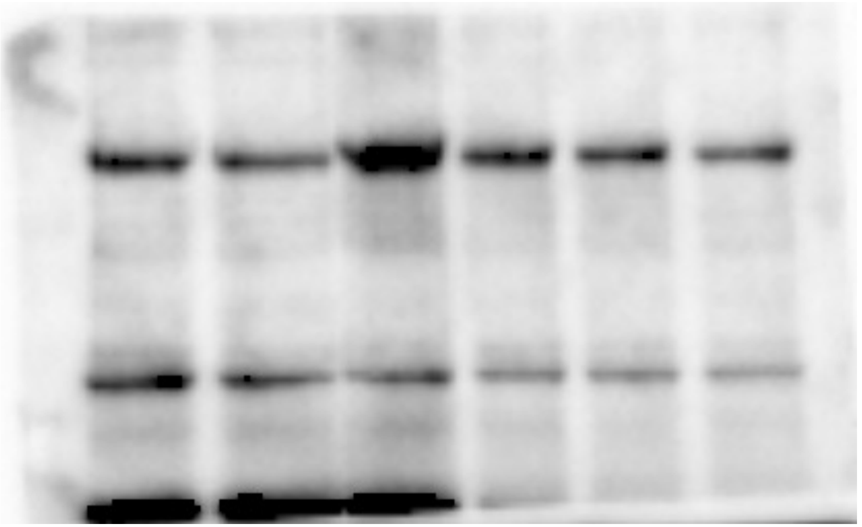

Full unedited gel for Figure 7D β-gal
